# Robust Generation of Oligodendrocyte Progenitors from Human Neural Stem Cells and Engraftment in Experimental Demyelination Models in Mice

**DOI:** 10.1371/journal.pone.0010145

**Published:** 2010-04-12

**Authors:** Margherita Neri, Claudio Maderna, Daniela Ferrari, Chiara Cavazzin, Angelo L. Vescovi, Angela Gritti

**Affiliations:** 1 San Raffaele Scientific Institute, San Raffaele Telethon Institute for Gene Therapy (HSR-TIGET), Milano, Italy; 2 Vita-Salute San Raffaele University, Milano, Italy; 3 Bioscience and Biotechnology Department, University of Milano-Bicocca, Milano, Italy; Julius-Maximilians-Universität Würzburg, Germany

## Abstract

**Background:**

Cell–based therapy holds great promises for demyelinating diseases. Human-derived fetal and adult oligodendrocyte progenitors (OPC) gave encouraging results in experimental models of dysmyelination but their limited proliferation *in vitro* and their potential immunogenicity might restrict their use in clinical applications. Virtually unlimited numbers of oligodendroglial cells could be generated from long-term self-renewing human (h)-derived neural stem cells (hNSC). However, robust oligodendrocyte production from hNSC has not been reported so far, indicating the need for improved understanding of the molecular and environmental signals controlling hNSC progression through the oligodendroglial lineage. The aim of this work was to obtain enriched and renewable cultures of hNSC-derived oligodendroglial cells by means of epigenetic manipulation.

**Methodology/Principal Findings:**

We report here the generation of large numbers of hNSC-derived oligodendroglial cells by concurrent/sequential *in vitro* exposure to combinations of growth factors (FGF2, PDGF-AA), neurotrophins (NT3) and hormones (T3). In particular, the combination FGF2+NT3+PDGF-AA resulted in the maintenance and enrichment of an oligodendroglial cell population displaying immature phenotype (i.e., proliferation capacity and expression of PDGFRα, Olig1 and Sox10), limited self-renewal and increased migratory activity *in vitro*. These cells generate large numbers of oligodendroglial progeny at the early stages of maturation, both *in vitro* and after transplantation in models of CNS demyelination.

**Conclusions/Significance:**

We describe a reliable method to generate large numbers of oligodendrocytes from a renewable source of somatic, non-immortalized NSC from the human foetal brain. We also provide insights on the mechanisms underlying the pro-oligodendrogenic effect of the treatments *in vitro* and discuss potential issues responsible for the limited myelinating capacity shown by hNSC-derived oligodendrocytes *in vivo*.

## Introduction

Development of therapeutic strategies to treat demyelinating diseases requires improved understanding of the molecular and epigenetic signals controlling the generation of mature oligodendrocytes from immature progenitors.

Short-term expandable cultures enriched in oligodendrocytes and glial-restricted progenitors have been generated from human embryonic neuroepithelial progenitors by means of epigenetic stimulation and immunophenotype selection [1], [2]. Also, subsets of oligodendrocyte progenitors (OPC) are separated from the adult human white matter based on their immunophenotype [3], [4]. These cells promote extensive myelination in models of experimental demyelination and congenital dysmyelination [4], [5], [6]. However, the limited OPC proliferation in vitro, which requires large amount of human donor tissue, and their potential immunogenicity might restrict their use in future clinical applications. In this view, improved understanding of the molecular and epigenetic signals controlling the generation of mature oligodendrocytes from self-renewing, multipotent neural stem cells (NSC) is crucial for the development of effective and feasible transplantation approaches and in view of the more challenging possibility to recruit endogenous progenitors for brain repair.

Continuous, non-transformed NSC lines have been established from the developing human (h) brain, either prospectively [7] or by epigenetic stimulation in floating cultures [8], [9], [10] and in adherent monolayers [11]. Human NSC display significant proliferation potential and amenability to viral-mediated gene-correction in vitro, coupled to lack of tumorigenicity upon grafting in the CNS, where they survive, migrate and differentiate appropriately [12], [13], [14], [15], [16], also delivering neuroprotective factors [17], [18], [19], [20]. Thus, they might provide a continuous supply of human neural precursors to be used for in vitro studies and for developing cell therapy approaches for neurodegenerative diseases of different origin [21], [22], [23]. However, in contrast to their rodent counterpart, hNSC give rise to small numbers of oligodendrocytes both in vitro [8], [24], [25], [26] and after transplantation in the CNS [27], [28], [29] [30].

Genetic and epigenetic in vitro manipulation of hNSC has been tested in order to obtain cell populations enriched in oligodendroglia. Viral-mediated over-expression of oligodendroglial genes [31] or immortalization of hNSC [32], [33] has allowed the generation of cell lines with appreciable oligodendroglial potential. In contrast, molecules able to promote proliferation, survival and maturation of rodent OPC from NSCs [34] are not similarly effective on hNSCs, pointing to inter-species differences in growth factor responsiveness [2], [35], [36]. Thus, additional studies aimed to define factors able to promote the enrichment and expansion of OPC from immature hNSCs are needed in the perspective of developing clinically applicable therapeutic strategies.

The aim of this work was to obtain enriched and renewable cultures of hNSC-derived oligodendroglial cells by means of epigenetic manipulation. We report here that large numbers of oligodendrocytes can be generated from long-term self-renewing hNSCs by a simple in vitro protocol based on concurrent/sequential in vitro exposure to combinations of growth factors (FGF2, PDGF-AA), neurotrophins (NT3) and hormones (T3). In particular, the combination FGF2+NT3+PDGF-AA resulted in the maintenance and enrichment of a proliferating oligodendroglial cell population displaying immature phenotype, limited self-renewal and increased migratory activity in vitro. These cells generate large numbers of oligodendroglial progeny at the early stages of maturation, both in vitro and after transplantation in models of CNS demyelination.

## Results

### Human NSCs express markers of immature oligodendroglial cells

**Figure 1:**
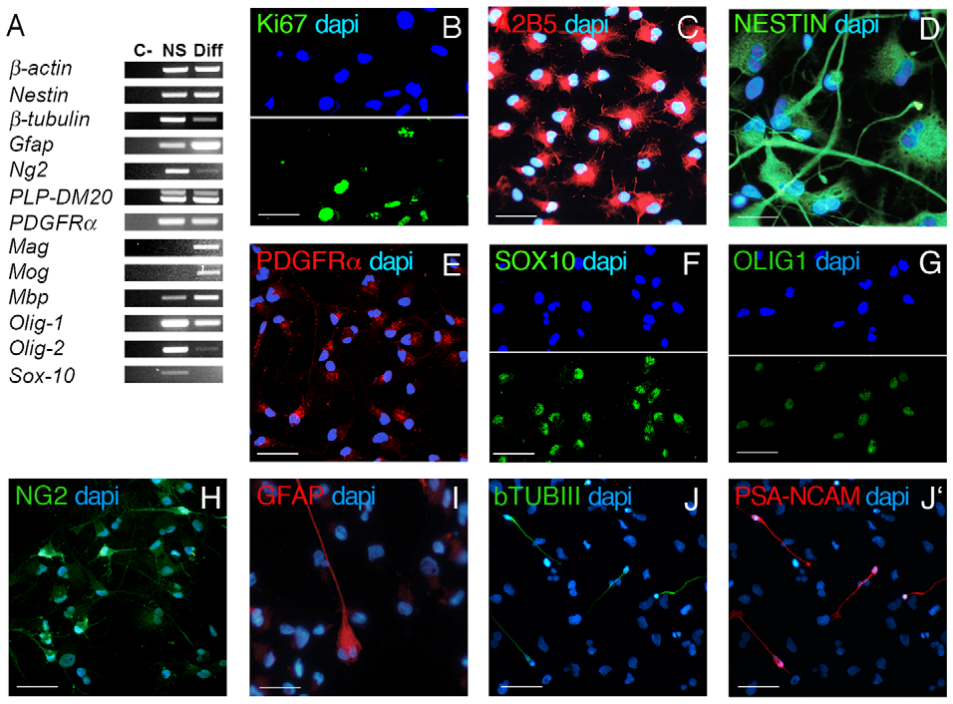
Human NSCs express oligodendroglial markers. (A) RT-PCR analysis showing down-regulation (β-tubulin III, Ng2, PDGFRα, Olig-1, Olig-2, Sox10) or up-regulation of transcripts (GFAP, Mag, Mog, Mbp) in hNSCs differentiated for 7 days in 2% FCS; Diff) compared to serially passaged hNSCs grown in EF medium (neurospheres, NS; C-, negative control). Cells dissociated from neurospheres were plated on an adhesive substrate in EF medium and analyzed by indirect immunofluorescence 24 hours later. Panels **B–H** show representative pictures of cells expressing proliferation markers (Ki67; **B**), markers of immature neuroepithelial cells (nestin; **D**) and oligodendroglial progenitors (A2B5, **C**; PDGFRα, **E**; Sox10, **F**; Olig1, **G**; NG2, **H**). Markers of mature neural cells: GFAP (**I**), β-Tubulin III and PSA-NCAM (**J** and **J'** represent the same field). Nuclei were counterstained with dapi. Scale bars: 30 µm (B, D, I), 50 µm (C, E, F, G, J, J').

We evaluated the presence in hNSC of molecules involved in the specification of the oligodendroglial lineage and in the proliferation, survival and differentiation of oligodendroglial precursors. Serially passaged neurospheres grown in EF medium express the mRNA for several markers of immature and mature oligodendroglial cells (Fig. 1A; NS). The presence of those markers was confirmed by immunofluorescence on cells dissociated from neurospheres and analyzed 24 hours after plating on an adhesive substrate in EF medium (precursor cells; Fig. 1B–J'). A large fraction of these cells expressed Ki67 (≈70%; proliferation marker; Fig. 1B), A2B5 (>90%; Fig. 1C) and nestin (>90%, Fig. 1D), markers of immature neuroepithelial cells and neural progenitors. A variable fraction of the hNSC population espressed PDGFα receptor (PDGFRα; 56.03±7.73%; Fig. 1E), Sox10 (64.77±5.79% Fig. 1F), Olig1 (58.71±8.59% Fig. 1G) and NG2 (19.73±8.49%; Fig. 1H), markers of rodent and human- oligodendroglial lineage cells [34], [36]. Interestingly, ≈80% of the NG2^+^ cells espressed PDGFRα. Also, all the PDGFRα^+^ cells expressed Olig1, while only 80% of them espressed Sox10. A minor proportion of the total cell population expressed the differentiation markers GalCer (0.93±0.05%; pre-myelinating oligodendrocytes; not shown), GFAP (astroglia; 2.27±0.54%; Fig. 1I), β-tubulin III and PSA-NCAM (3.01±1.10%; neurons; Fig. 1J,J'). The distribution and expression pattern of PSA-NCAM and β-tubulin III completely overlapped.

In order to drive progressive lineage restriction and cell differentiation precursor cells were washed free of mitogens and exposed to medium containing 2% FCS for 7 days. In differentiated cultures (Fig. 1A; Diff) we found increased mRNA levels for astroglial (GFAP) and oligodendroglial (MAG, MOG, MBP) markers, paralleled by down-regulation of markers highly expressed in precursors (i.e. NG2, PDGFRα, Olig2 and, to a lesser extent, Olig1 and Sox10). On the contrary, mRNA levels for PLP-DM20 (a major myelin protein), nestin and β-tubulin III were similar or lower to those observed in precursors. Immunofluorescence analysis largely confirmed RT-PCR data, showing increased numbers of astroglia (GFAP; 60.16±5.31%) and neuronal cells (TUJ1; 6.60±3.29%), decreased proliferation activity (30.91±7.17 and 16.42±3.19 Ki67^+^ and PCNA^+^ cells, respectively) and reduced numbers of oligodendrocyte progenitors (NG2^+^, O4^+^ and PDGFRα^+^ cells represented 4.83±1.05%, 0.59±0.51%, and 9.42±3.11% of the total cell population, respectively). GalCer was present in 2.49±0.46% of the cells. Nestin expression was maintained in ≈50% of GFAP^+^ cells but was not observed in TUJ1^+^ or GalCer^+^ cells. Expression of Sox10 and Olig1 was maintained in 34.01±9.67% and 53.79±13.29% of the total cell population, respectively. Interestingly, a relevant fraction of the PDGFRα^ +^, Sox10^+^ and Olig1^+^ cell population in the differentiated cultures still expressed proliferation markers (24.63±3.26% Ki67^+^ PDGFRα^+^, 21.54±2.3% PCNA^+^Sox10^+^ and 19.11±2.44% PCNA^+^Olig1^+^ cells).

These data indicated that removal of mitogens and addition of serum (a protocol that we call hereafter *default differentiation protocol*) drives hNSC mainly towards the astroglial lineage. They also point out that hNSCs contain a relevant population of proliferating putative oligodendroglial progenitors.

### Selected extrinsic signals direct hNSCs into oligodendrocyte progenitors

**Figure 2:**
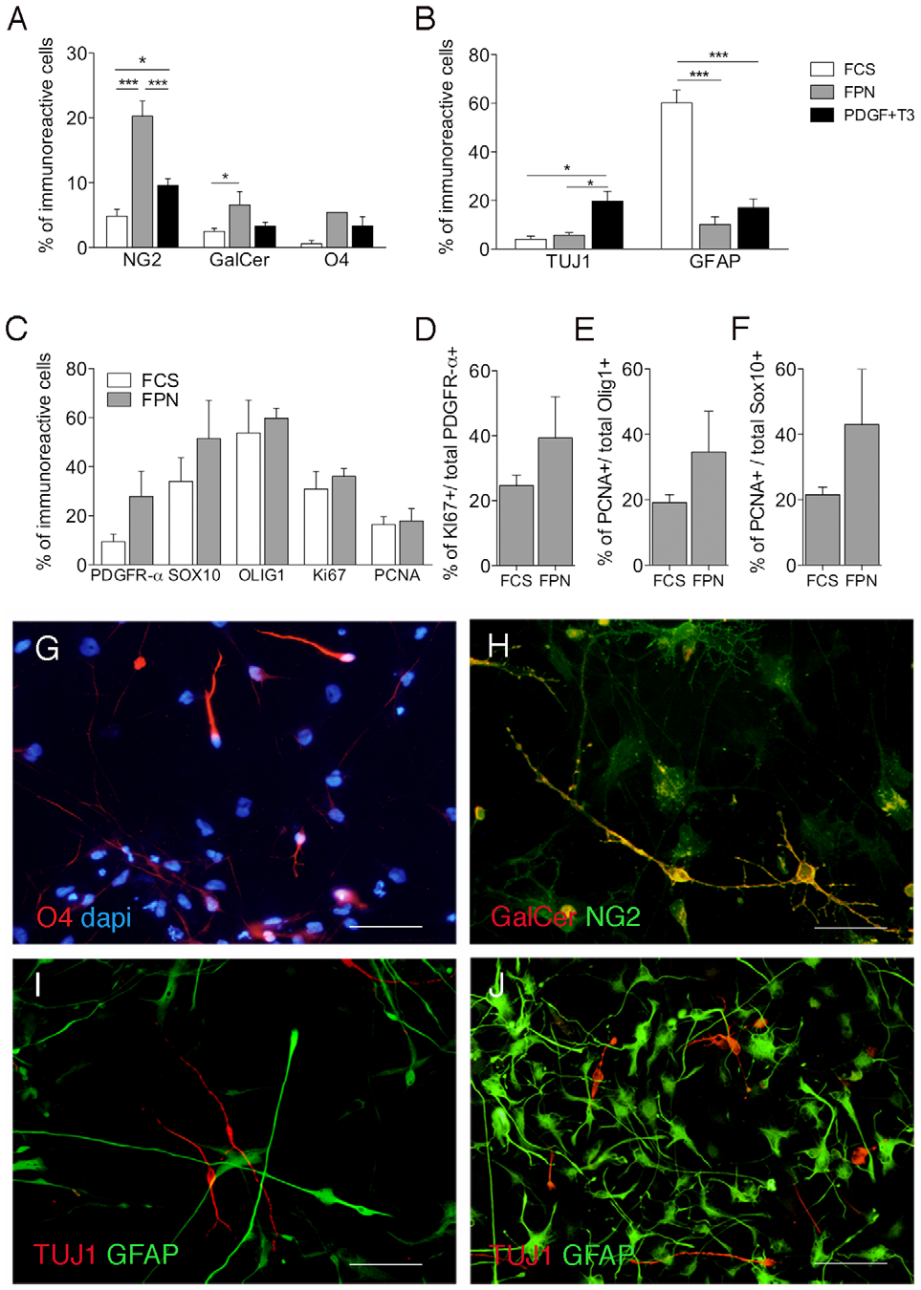
Selected epigenetic signals drive hNPCs to become oligodendrocytes in vitro. (**A, B**) Exposure of hNSCs to FPN or PDGF-AA+T3 increased the numbers of NG2^+^, O4^+^ GalCer^+^ oligodendroglil cells (**A**) and TUJ1^+^ neuronal cells (**B**), while decreasing the numbers of astroglial cells (**B**) compared to the default differentiation protocol (FCS). (**C**) Increased percentages of PDGFRα^+^ and Sox10^+^ cells are present in FPN-treated cultures as compared to FCS-treated cultures. (**D–F**) FPN-treated cultures contain twice as much proliferating cells (Ki67^+^) within the putative oligodendroglial progenitor cell population (PDGFRα, **D**; Olig1, **E**; Sox10, **F**) as compared to FCS-treated cultures. (**G–H**) Immature morphology of O4^+^ cells (**G**) and co-expression of NG2 and GalCer in FPN-treated cells (**H**; orange colour represent merged signal). (**I, J**) Representative pictures of neurons (TUJ1) and astrocytes (GFAP) in FPN (**I**) and FCS-treated (**J**) cultures. Data in **A–F** are expressed as the mean±SEM, n = 2−4 independent experiments, 3–17 total replicates. Data were analyzed by Two-way ANOVA followed by Bonferroni posttests. ***p<0.001,*p<0.05. Scale bars: 80 µm (G, I, J); 50 µm (H).

We next checked whether removal of FCS from the medium and addition of growth factors (FGF2, PDGF-AA), hormones (T3) and neurotrophins (NT3) known to support proliferation, survival and differentiation of rodent's OPC could improve the yield of hNSC-derived oligodendrocytes. The first treatment was FGF2+PDGF-AA+NT3 (10, 20 and 20 ng/ml, respectively - FPN medium) and the second was PDGF-AA+T3 (20 and 40 ng/ml, respectively). Serially passaged neurospheres were washed free of EF medium and mechanically dissociated. Cells were plated in the different treatments for 7 days, then evaluating the percentage of different cell types in the cultures by immunocytochemistry. Treatment with either combinations resulted in increased percentages of NG2^+^, O4^+^ and GalCer^+^ cells in hNSC-derived cultures compared to the default differentiation protocol (FCS; Fig. 2A) and this was paralleled by a 5-fold reduction in the number of astrocytes (Fig. 2B; I, J). FPN treatment was more effective than PDGF treatment in generating oligodendroglial cells. Thus, we decided to investigate in more detail the presence of oligodendroglial progenitors in FPN-treated cultures. This analysis revealed an increase in the number of PDGFRα^+^ cells and Sox10^+^ cells as compared to FCS-treated cultures (Fig. 2C). Although the total percentage of cells expressing proliferation markers was similar in the two treatments (≈35% and 16% of Ki67^+^ and PCNA^+^ cells, respectively; Fig. 2C), exposure to FPN increased of about two-fold the percentage of proliferating cells in the putative oligodendroglial progenitor cell population (PDGFRα^+^, Olig1^+^, Sox10^+^ cells; Fig. 2D–F).

This result, coupled to the immature morphology of O4^+^ (Fig. 2G) and GalCer^+^ cells (Fig. 2H) and to the persistence of NG2 expression in 20–50% of GalCer^+^ cells (Fig. 2H) indicated that a short treatment with FPN medium maintained and expanded a population of putative oligodendrocyte progenitors that could eventually differentiate in mature oligodendrocytes if exposed to additional treatments.

A schematic of the qualitative and quantitative results for the different culture conditions is shown in Fig. S1.

### FPN priming increases the yield of hNSC-derived oligodendrocytes in differentiated cultures

**Figure 3:**
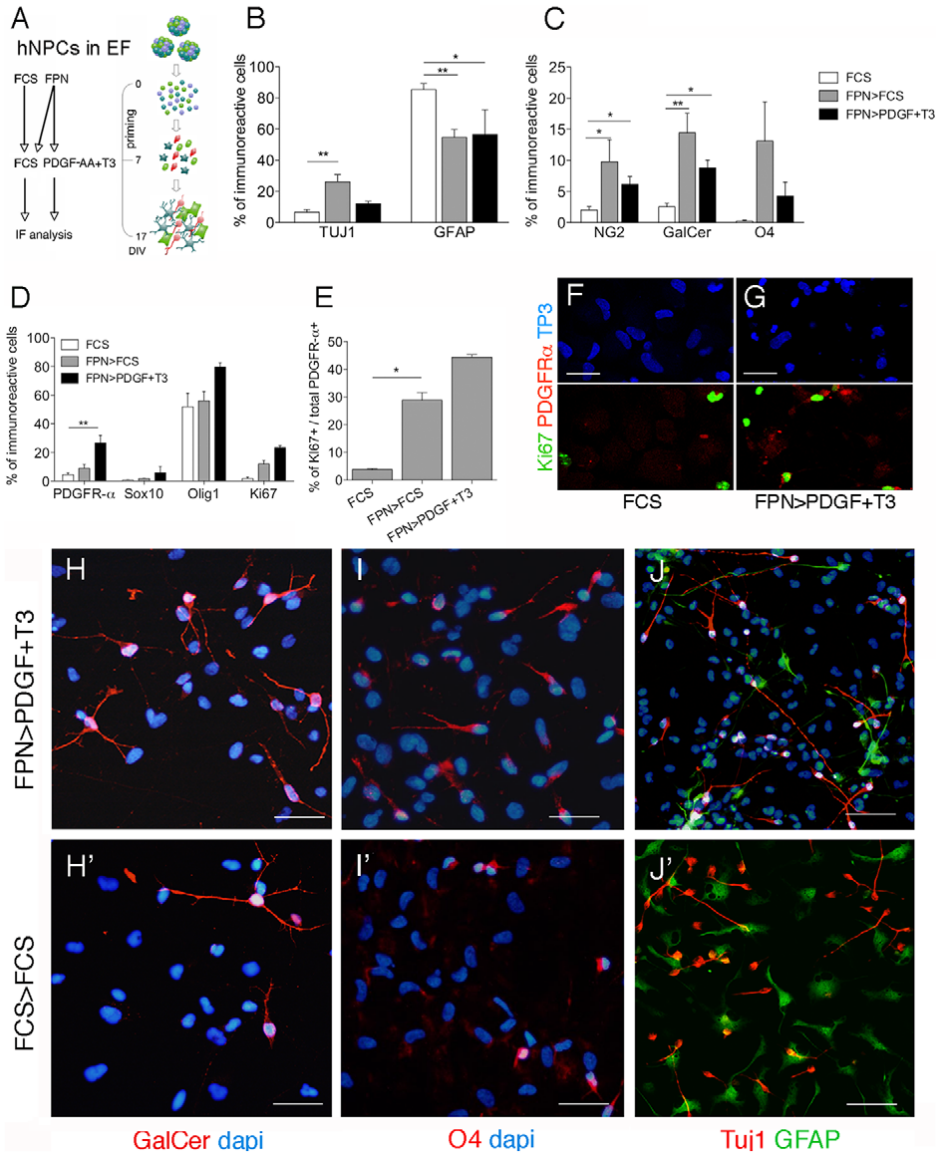
Priming with FPN medium increases the yield of oligodendrocytes. (**A**) Scheme summarizing the experimental protocol. (**B–C**) FPN-primed cultures showed decreased numbers of astrocytes (GFAP) and increased numbers of neurons (TUJ1) compared to FCS-primed cultures (**B**) as well as a 3–6 fold-increase in the number of NG2-, GalCer- and O4-expressing cells (**C**). (**D**) Percentages of cells expressing markers of oligodendrocyte precursor and.proliferating cells in the different treatments. (**E**) Increased fractions of KI67^+^ cells within the PDGFRα^+^ cell population in the FPN-primed cultures (either FCS- or PDGF-AA+T3-treated). Data are expressed as the mean±SEM, n = 2−4 independent experiments with 3–12 total replicates. Comparisons between treatments were performed for individual antigens using Kruscal Wallis test followed by Dunn's multiple comparison test ***p<0.001, **p<0.01. (**F, G**) Representative confocal pictures of proliferating (Ki67, green) PDGFRα^+^ (red) cell in FCS-(**F**) and FPN>PDGF+T3-treated cultures (**G**). Nuclei counterstained with ToPro3 are shown separately (TP3; blue). (**H–J'**) Representative immunofluorescence pictures showing the morphology of oligodendroglial cells (GalCer, **H–H'**; O4, **I–I'**), neuronal and astroglial cells (TUJ1 and GFAP; **J–J'**) in FPN-primed (FPN>PDGF-AA+T3; **H–J**) compared to FCS treated cultures (FCS>FCS; **H'–J'**). Scale bars: 30 µm (F, G), 40 µm (H–I'), 100 µm (J, J').

In order to assess whether the enrichment in oligodendroglial progenitors obtained by FPN treatment could be actually exploited to increase the yield in mature oligodendrocytes, hNSCs grown in FPN medium for 7 days were shifted to FCS or PDGF-AA+T3 (known as pro-differentiating signals for rodent and human OPCs) and grown for additional 10 days. Cells grown for 17 days in FCS were used as control. Cultures were then analyzed for the presence of neurons, astrocytes and oligodendroglial cells (Fig. 3A).

The additional 10-day differentiation period resulted in an overall decrease of proliferation activity, reduced numbers of immature progenitors and increased numbers of cells expressing lineage-specific markers with respect to the values described in 7-day treated cultures. In FPN-primed cultures we detected reduced numbers of GFAP^+^ cells and increased numbers of TUJ1^+^ cells as compared to FCS-primed cultures (Fig. 3B, J–J'), together with a 3- to 6-fold increase in the percentages of NG2^+^, O4^+^ and GalCer^+^ cells, which reached 10–20% of the total number of cells. This effect was more robust in FPN>FCS cultures as compared to FPN>PDGF-AA+T3 cultures (Fig. 3C; Fig. S1). The decrease in the percentage of KI67^+^ cells observed in FCS>FCS cultures (from 30% at 7DIV to 2% at 17 DIV) was partially or totally prevented in FPN>FCS and FPN>PDGF-AA+T3 cultures, respectively. A similar effect was observed on the PDGFRα^+^ cell population (Fig. 3D). The presence of higher proportions of KI67^+^ cells within the PDGFRα^+^ cell population in the FPN-primed cultures (either FCS- or PDGF-AA+T3-treated) further confirmed the specific effect of this sequential treatment in expanding and maintaining a population of hNSC-derived oligodendroglial progenitors (Fig. 3E–G; a schematic of the culture conditions and the percentages of cells immunoreactive for lineage-specific and proliferation markers are summarized in Fig. S1).

This conclusion was further supported by immunofluorescence analysis showing O4^+^ cells displaying uni- and bipolar morphology typical of oligodendrocyte progenitors (Fig. 3I, I'). The GalCer^+^ cell population (Fig. 3H, H') was morphologically heterogeneous, and included bipolar cells and cells displaying features of non-myelinating oligodendrocytes. A variable proportion of GalCer^+^ and O4^+^ cells co-expressed NG2 (Fig. S2A,B). Importantly, NG2, GFAP and PSA-NCAM expression were mutually exclusive in hNSC-differentiated culture and identified oligodendroglial precursors, astroglia and cells of the neuronal lineage, respectively, regardless the differentiation protocol used (Fig. S2C–F). We did not detect MBP^+^ cells, suggesting that these differentiating conditions were not sufficient to drive a substantial shift from oligodendrocyte progenitors and pre-myelinating oligodendrocytes to mature oligodendrocytes.

### Human NSC-derived oligodendrocyte precursors expanded as neurospheres in FPN medium

**Figure 4:**
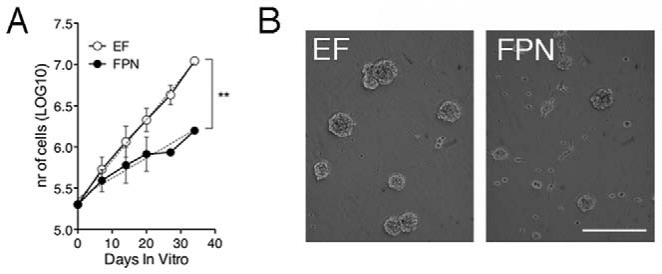
hNSCs grown in FPN medium show limited expansion and self-renewal. (**A**) Growth curves were established for EF and FPN hNSCs as described in Methods. Each value point of the curves represents the mean±SEM of three independent experiments. Data were interpolated using a linear regression model and best fitted the following equation: *y*  =  *a* + *b*x, where *y* is the LOG10 of the total number of cells, x is the time (DIV), a is the intercept, and b is the slope. Values of b±SEM are 0.04948±0.00495 (EF) and 0.02447±0.00512 (FPN) **p = 0.017. Dotted lines represent regression lines. (**B**) Human NSCs in EF medium generated typical floating neurospheres, while FPN-treated hNSCs showed small clusters and isolated bipolar cells adhering to the flask. Scale bar, 200 µm.

Based on the results described above and with the aim of generating a renewable source of hNSC-derived OPCs we decided to test whether exposure of undifferentiated hNSCs to FPN medium could expand human-derived oligodendrocyte precursors in neurosphere cultures. We collected hNSCs grown in EF medium and plated them in EF or in FPN medium, establishing growth curves in the two different conditions. Culturing hNSCs in FPN medium significantly reduced their growth rate, still maintaining their self-renewing capacity for at least 5 subculturing passages (Fig. 4A). While hNSCs in EF medium generated typical floating neurospheres the FPN-treated hNSCs showed a large proportion of small clusters and isolated cells adhering to the flasks and showing the morphology of migrating progenitors (Fig. 4B).

**Figure 5:**
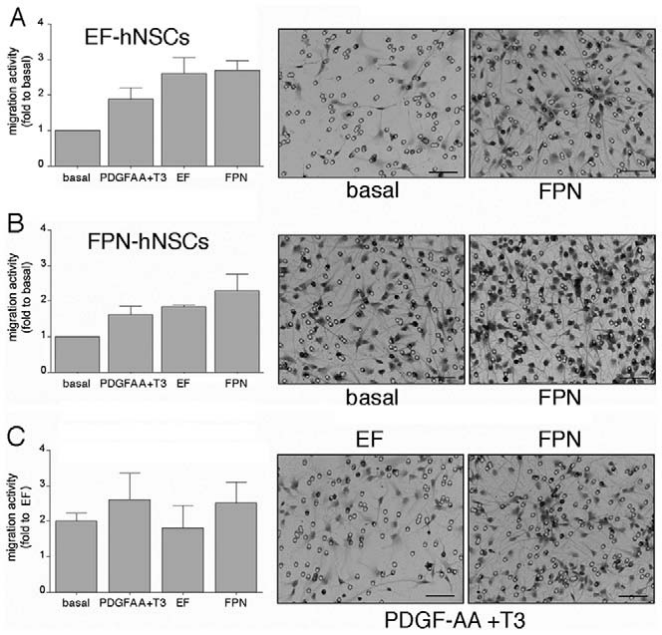
FPN-hNSCs show increased migratory activity. (**A, B**) EF- and FPN-hNSCs display a 2–3 fold increase in migratory activity in response to the different treatments. (**C**) FPN hNSCs display a 2–3 fold increase in migratory activity compared to EF-hNSCs, both in control medium and in response to the different treatments. Pictures show representative fields for selected experimental conditions. Scale bars: 40 µm. Data are expressed as the mean ± SE and derive from 2–4 independent experiments, 1–2 replicates for each experiment.

We tested the migratory activity of FPN- and EF-hNSC populations either in basal medium or in the presence of different culture conditions (EF, FPN and PDGF-AA+T3). Our data showed that all the treatments trigger a 2–3 fold increase in migratory activity in both cell populations as compared to the basal condition (Fig. 5A, B). In order to test the potential “intrinsic” difference in migratory activity of the two cell populations, the migratory activity of FPN hNSCs was espressed as fold increase to the migratory activity of EF hNSCs in each culture condition. By doing so, we demonstrated that FPN-hNSCs displayed a consistent 2–3 fold increase in migratory activity compared to EF-hNSCs, both in basal media and in response to the different treatments (Fig. 5C).

**Figure 6:**
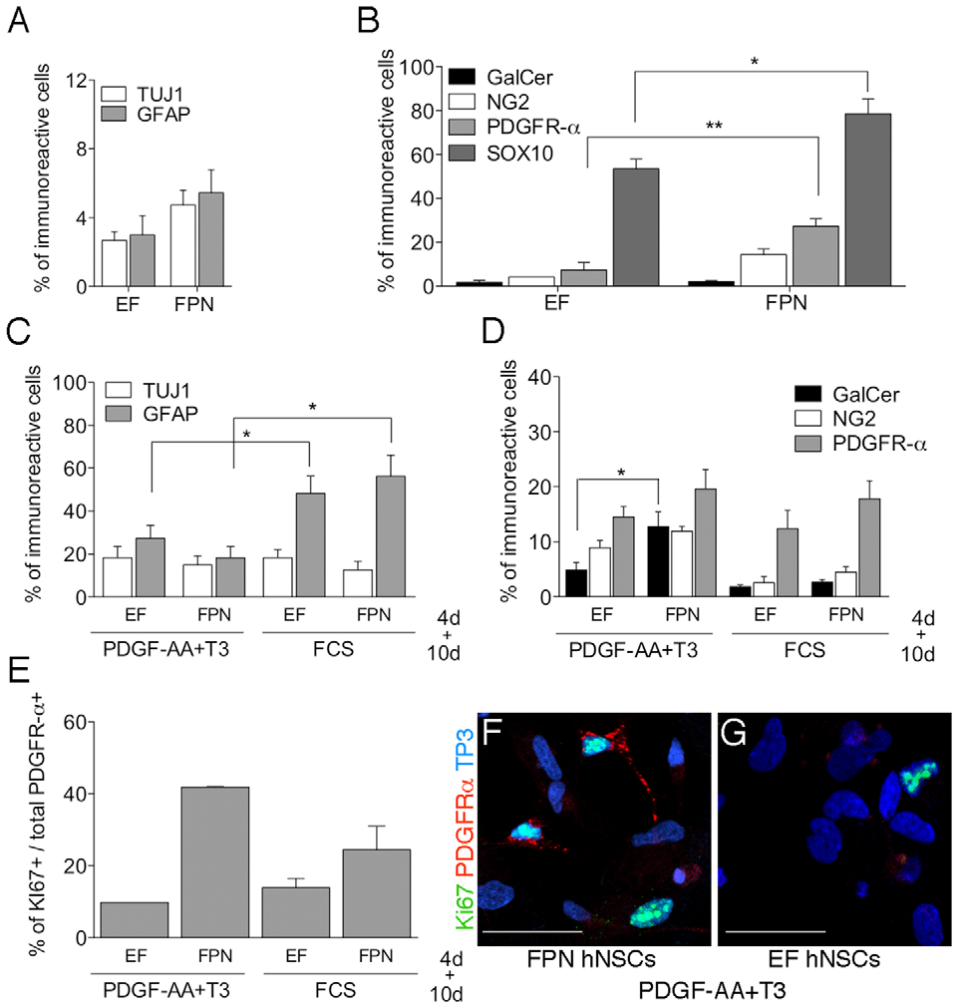
Myelinogenic potential of FPN hNSCs in vitro. (**A–B**) Percentages of neurons (TUJ1), astrocytes (GFAP; **A**) and oligodendrocytes (NG2, PDGFRα and Sox10; **B**) in FPN- compared to EF-treated hNSCs four days after plating in their respective culture media. (**C**) Increased percentages of TUJ1^+^ and GFAP^+^ cells at the end of the differentiation period (14 days) in FCS or PDGF-AA+T3 (**D**) Percentages of GalCer^+^ cells in PDGF-AA+T3-treated cultures derived from FPN- and EF- hNSCs (14 days). (**E**) Moderate increase in the total number of PDGFRα^+^ cells and higher proportion of Ki67^+^PDGFRα^+^ in FPN-derived hNSCs in comparison to the EF-derived counterpart. (**F, G**) Representative merged confocal pictures showing PDGFRα^+^ cells (red) expressing Ki67 (green) in FPN- (**F**) and EF-hNSCs (**G**) differentiated in the presence of PDGF-AA+T3. Nuclei counterstained with ToPro3 (blue). Data are expressed as the mean±SEM, n = 3−4 independent experiments, 2–16 total replicates. Comparisons between the two cell populations were performed for different treatments and for individual antigens using Mann-Whitney test. **p<0.01, *p<0.05. Scale bars: 30 µm (F, G).

Finally, hNSCs grown for 5 passages in either FPN or EF medium were differentiated by plating in FPN and EF medium, respectively (4 days), and subsequently exposing them to FCS or PDGF-AA+T3 for additional 10 days. After 4 days (Fig. 6A, B) we found increased percentages of neurons and astrocytes (Fig. 6A), higher numbers of NG2^+^, PDGFRα^+^ and Sox10^+^ cells and similar numbers of GalCer^+^ (<3%) (Fig. 6B) and Olig1 (≈70%; not shown) in FPN- compared to EF-derived cultures. Interstingly, ≈80% of NG2^+^ cells co-expressed PDGFRα. This overlap suggests that, under these culture conditions, NG2 and PDGFRα co-expression identify OPCs developing from hNSCs. Both the absolute percentage of Ki67^+^ cells and the percentage of Ki67^+^ cells within the PDGFRα^+^ cell population were similar in EF- and FPN hNSCs at this early time-point (≈40%). Culturing either EF and FPN hNSCs for additional 10 days in either PDGF-AA+T3 or FCS promoted their overall neuronal and astroglial differentiation, as shown by increased percentage of TUJ1^+^ and GFAP^+^ cells, the latter being reduced in PDGF-AA+T3 as respect to FCS-treated cultures (Fig. 6C). In the presence of PDGF-AA+T3 we found increased numbers of GalCer^+^ in FPN-derived differentiated cells with respect to their EF-derived counterpart (Fig. 6D). Similar proportions of Olig1^+^ (≈50–60%) and Sox10^+^ cells (≈10%) were maintained (not shown), regardless the treatment, and only a slight increase in the proportions of the total PDGFRα^+^ cells were observed in FPN-derived differentiated cells (Fig. 6D). However, the fraction of proliferating cells within the PDGFRα^+^ cell population was higher in FPN-derived hNSCs differentiated either in FCS of in the presence of PDGF-AA+T3 (Fig. 6E–G).

These data indicated that growing hNSCs as neurospheres in FPN medium allows the enrichment and expansion of an oligodendroglial progeny, a relevant proportion of which retains an immature phenotype when exposed to our differentiation protocol. These results were further supported by: i) the presence of increased proportions of Olig1^+^ PDGFRα^+^ (≈20% versus 8%; Fig. S3A) and, to a lesser extent, of Sox10^+^PDGFRα^+^ cells (≈6% vs 2%; Fig. S3B) in FPN vs EF hNSCs exposed to PDGF-AA+T3; ii) the presence of variable proportions of PDGFRα^+^ hNSCs expressing nestin (Fig. S3C), but not PSA-NCAM (Fig. S3D) or GFAP (Fig. S3E, F).

In our quantification experiments we provided all results as percentages. In order to rule out the possibility that increased percentages of putative OPCs might results from different proliferation behavior of different cell populations depending on the medium used, we analyzed the absolute numbers of cells and the number of cells positive for each specific antigen used to identify putative OPC, obtaining results consistent with our original interpretation. Examples of absolute number analysis are shown in Table S3.

When we applied the FPN priming paradigm to cortex (CTX)-derived hNSCs [8], we found similar results in terms of proliferation and differentiation capacity towards the oligodendroglial lineage. However, GalCer^+^ oligodendrocytes generated from CTX-derived FPN hNSCs cells consistently displayed a more mature morphology (multiple branched processes and myelin-sheet like formations) with respect to those generated by telencephalon-derived FPN hNSCs cells (Fig. S4).

### Myelinogenic competence of hNSCs in a model of focal demyelination

**Figure 7:**
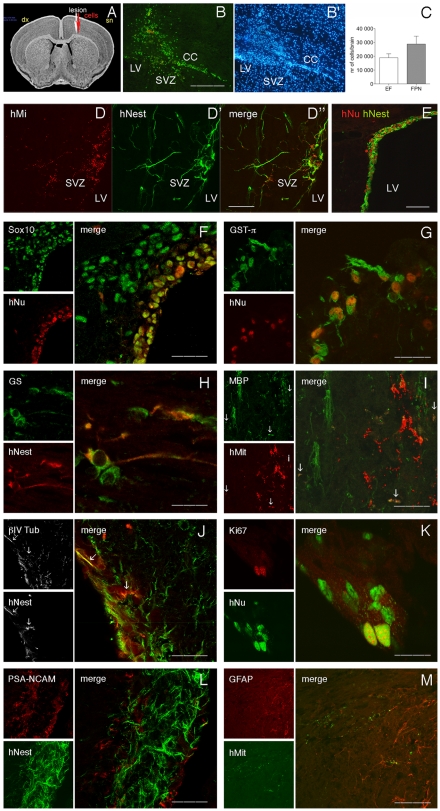
Myelinogenic competence of hNSCs in a focal model of demyelination. (**A**) EF- or FPN-hNSCs (200.000 cells/animal) were transplanted at the lesion site 48 h after lysolecithin injection. (**B–C**) Engrafted hNSCs (hNu; **B**) were present close to the injection site, in the corpus callosum (CC) and in the subventricular zone (SVZ) of the lateral ventricles (LV) two months after injection. Nuclei counterstained with dapi (**B'**). (**C**) Quantification of engrafted cells in mice transplanted with FPN- and EF-hNSCs. Data are expressed as the mean number of cells/brain ±SEM (n = 5 animals/group; unpaired Student t test, p = 0.07). (**D–E**) Double-labelling immunofluorescence using antibodies recognizing human Mitochondria (hMit) and human Nestin (hNest; **D–D”**) or hNu and hNest (**E**) showed overlapping distribution. (**F–M**) Engrafted hNSCs expressed nestin (**H, J, L**), Sox10 (**F**), βIV Tubulin (early oligodendroglial marker; **J**), GST-π (marker of mature oligodendrocytes; **G**). Few (<2%) human-derived cells expressed MBP (**I**, arrows) and astrocytes markers (Glutamine synthase – GS, **H**; GFAP, **M**). We did not detect human cells expressing neuronal markers (PSA-NCAM, **L**). Scattered hNSCs in the SVZ expressed the proliferation marker Ki67 (**K**). Scale bars: 200 µm (E), 100 µm (B, B', F, J, L, M, I), 50 µm (D–D”), 25 µm (G, H, K).

We tested the ability of EF- and FPN-hNSCs to engraft and differentiate following transplantation in a focal model of demyelination induced with a single injection of lysolecithin in the corpus callosum of adult mice. In this model, demyelination occurs in about 48 hours and reaches maximum levels after 4–5 days. EF- or FPN-hNSCs were transplanted in the lesion site 48 h after lysolecithin injection (Fig. 7A). A consistent fraction of hNSCs (10–14% of the total number of transplanted cells), detected by means of antibodies that specifically recognizes a human nuclear protein (hNu), engrafted and survived up to 8 weeks post-transplantation (the longest time point analyzed) and were found in regions close to the injection site, in the corpus callosum and in the subventricular zone (SVZ) of the lateral ventricles (Fig. 7B, B'). We observed higher numbers of engrafted cells in the FPN hNSC-treated with respect to the EF hNSC treated group (Fig. 7C) but no differences in rostro-caudal cell distribution was observed between the two groups (3.917±295 µm and 4.073±247 µm for EF and FPN hNSCs, respectively). Human NSCs were also detected by using antibodies that specifically recognized hMitochondia (hMit) and hNestin (hNest; Fig. 7D–D''), or hNu and hNest (Fig. 7E), which revealed overlapping expression patterns. The engrafted hNSCs displayed variable morphology, with cell processes extending in the brain parenchima. The majority of them (>80%) expressed nestin (Fig. 7H, J, L) and vimentin (not shown), markers of neuroepithelial cells and immature glia.

Double labelling immunofluorescence using antibodies against lineage-specific markers showed that relevant proportion of engrafted hNSCs (>80%) expressed Sox10 (Fig. 7F) and Olig1 (not shown), suggesting the maintenance of the oligodendroglial potential shown *in vitro*. A small fraction of hNSCs expressed βIV-Tubulin, a marker of early oligodendroglial cells [37], [38] (Fig. 7J, arrows). Most important, about 15% of engrafted hNSCs expressed Glutathion-S-Transferase-*π* (GST-*π*; Fig. 7G), a marker of mature oligodendrocytes in rodents [39]. Only a small percentage of human-derived cells expressed MBP (<2%; Fig. 7I) or the astrocytic markers Glutamine synthase (GS, Fig. 7H) and GFAP (Fig. 7M). We did not detect hNSCs expressing neuronal markers (TUJ1, not shown; PSA-NCAM, Fig. 7L). We found few cells of donor origin expressing the proliferation marker Ki67 localized along the lateral ventricle (Fig. 7K). We did not find relevant differences in the proportions of the different cell types among the engrafted FPN- and EF- hNSCs.

In order to further strenghten these observations we transplanted EF and FPN- hNSCs in the corpus callosum of young Shiverer mouse, a model in which the lack of endogenous Mbp allows an easier and more direct read-out of the myelinogenic competence of the transplanted cells. Two months after transplant we observed significant engraftment and migration of hNSCs, the majority of which maintained nestin expression (Fig. S5). Similar results were observed in mice analyzed 3 and 4 months after transplant (data not shown).

## Discussion

We describe here a simple and reliable method to generate large numbers of oligodendroglial progenitors from a renewable source of somatic, non-immortalized hNSCs by exposure to a specific combination of growth factors, neurotrophins and hormones. Our data clarify the mechanisms underlying the pro-oligodendrogenic effect of the treatments in vitro and point to critical issues that have to be considered when evaluating the myelinogenic competence of hNSC-derived oligodendrocytes in experimental models of CNS demyelination developed in mice.

Oligodendroglial cells express a set of growth factors receptors, proteoglycans and transcription factors, according to their stage of lineage progression. We provide evidence that, similarly to glial restricted progenitors [2], [4] and OPCs [40], the vast majority of hNSCs are negative for the polysialilated form of the neural cell adhesion molecule (PSA-NCAM), the expression of which in our hands is restricted to cells of the neuronal lineage. Instead, hNSC are positive for the A2B5 ganglioside-specific antigen (>90%) and for the PDGFRα (>50%). A minor proportion (≈20%) express high levels of the chondroitin sulphate proteoglycan NG2, a marker of intermediate and late OPCs and early pre-oligodendrocytes [40], and the majority of these cells co-express PDGFRα, indicating overlap of these two markers in hNSC-derived OPCs. In addition, ≈60% of undifferentiated hNSCs express the transcription factors Olig1 and Sox10, which are widely expressed in the oligodendroglial lineage and play fundamental roles in the differentiation and maintenance of CNS oligodendroglial populations during development and in adulthood, in physiological conditions and in disease [41], [42], [43], [44]. The proportions of the different cell types was stable over time (from passage 18 to 25), indicating that relevant percentages of cells within the serially passaged hNSC bulk population grown as neurospheres display a molecular and immunochemical signature closely resembling that described for OPC populations isolated from the human CNS by means of immunopanning or FACS sorting [4], [5], [45].

Removal of mitogens (either with the addition of low serum concentration or in serum-free conditions) results in the generation of large numbers of oligodendrocytes from embryonic and post-natal murine NSC lines (10–20% GalCer^+^ and O4^+^ cells) [25], [34], [46] [47]. In contrast the yield of oligodendrocytes generated from foetal hNSCs using similar differentiation protocols is either not reported (directly isolated hNSCs)[7] or shown to be low (epigenetically isolated hNSCs; <3% of the total number of cells in culture) [8], [25], [26], [35], [36], [47], [48]. The down-regulation of mRNA levels for PDGFRα and Olig1/2 and Sox10 genes, coupled to up-regulation of myelin-specific genes in hNSC cultures that we observed applying the *default differentiation protocol* argued against the idea of reduced oligodendroglial potential of human versus murine NSCs. However, the lack of robust expression of markers of foetal and adult human pre- and early oligodendrocytes (i.e. O4, GalCer)[36], [40], [49], [50], [51] or myelinating oligodendrocytes (Mbp, Plp)[5] pointed to the incapability of this treatment in sustaining either survival, proliferation or lineage progression of human OPCs. Several reports indicate differences in growth factor responsiveness between rodent and hNSCs as regard to oligodendroglial production [2], [35], [36]. At the same time, FGF2, PDGF-AA and neurotrophin 3 (NT3), molecules that control rodent OPC proliferation, survival and migration in vitro [34], [52], [53], [54] and in vivo [55], [56], are shown to promote proliferation of spinal cord- [40] and white matter-derived human OPC [4]. These observations suggest that murine and hNSCs might share responsiveness to a set of growth factors that control lineage specification, OPC survival and proliferation, envisaging species-specific differences regarding the timing of differentiation in response to these factors.

We found that a 7 day-exposure of hNSCs to FPN medium increased the proportion of O4- and GalCer-expressing oligodendrocytes to 15–20% of the total number of cells in culture, a yield that has never been reported so far from naïve hNSC. Interestingly, we could maintain hNSCs as neurospheres in FPN medium for at least 5 subculturing passages, achieving a moderate but still important expansion of the cell population (10-fold versus 100-fold expansion in FPN- versus EF hNSCs). The morphology of FPN-treated hNSCs, their increased migratory activity, the increased percentages of proliferating cells expressing early OPC markers and the reduced percentages of astrocytes in differentiated cultures strongly support an effect of FPN priming in promoting the expansion or survival of hNSC-derived OPCs at the expenses of astroglial and, possibly, neuronal progenitors. However, we cannot rule out the possibility of a treatment-induced lineage shift (similar to that played by IGF-1 on murine NSCs) [57]. A detailed comparative analysis of proliferating neuronal and glial population is needed to address this issue.

A growth factor can mediate cell proliferation, survival or differentiation depending on the stage of CNS development and/or cell differentiation. The PDGF/PDGFRα signaling pathway provides mitogenic and migratory signals in rodent [52], [58] and human [40] early OPCs, while later in development it has a major survival effect [59]. The sequential and independent roles of PDGF in proliferation and survival are mediated by PDGF-mediated integrin activation, with α6β1 integrin playing a major role [60], [61], [62]. We have recently shown that the vast majority of hNSCs express a panel of adhesion molecules, among which α2, α6, and β1integrins are the most represented [63], thus suggesting a role for this signalling pathway in hNSCs and in their progeny. Our data showing that exposure of FPN-primed cultures to PDGF-AA is more efficient in delaying oligodendrocyte differentiation compared to FCS (as shown by decreased percentages of NG2-, GalCer- and O4-expressing cells coupled to a moderate increase in PDGFRα^+^Ki67^+^ cells) are consistent with all these notions. Thyroid hormone is key signal in brain development, oligodendrocyte development and myelin protein gene expression regulation [64], [65] and can regulate oligodendroglial lineage and maturation in neurospheres derived from the subventricular zone of adult rats [66]. Similar to PDGF, it may act at multiple steps in the development of oligodendrocytes [67]. Additional investigation is needed to determine whether its effect on hNSCs is additive or synergic to PDGF.

The large number of oligodendrocytes generated from hNSCs did not reach the immunophenotypic features of myelinating cells Interestingly, GalCer^+^ oligodendrocytes generated from CTX-derived FPN hNSCs cells consistently displayed a more mature morphology (multiple branched processes and myelin-sheet like formations) with respect to those generated by telencephalon-derived FPN hNSCs cells. This suggests that the region-dependent ability described for both rodent [46], [47] ad human-derived NSCs [48] to differentiate along the neuronal and glial lineage might apply to the oligodendroglial lineage as well. Exposure of FPN-primed cultures to BDNF, CNTF or IGF1, molecules known to exert a maturational effect on rodent [68] and human OPCs [40] resulted in more mature morphology of hNSC-derived oligodendrocytes, still without achieving MPB expression (A.Gritti, unpublished results). Additional differentiation steps and a prolonged time window of in vitro differentiation using these or other molecules, alone or in combination, might be necessary to push the maturation of hNSC-derived oligodendrocytes.

Our results indicate that hNSCs transplanted in a model of focal CNS demyelination integrate, migrate and differentiate along the oligodendroglial lineage. While higher numbers of engrafted cells were observed in the FPN-transplanted group, the proportions of EF- and FPN hNSC-derived Sox10-, Olig1- and GST-π-expressing cells were similar. In addition, a very low yield in MBP^+^ cells was found in both groups. These results are in apparent contrast with our in vitro data showing improved ability of oligodendrocyte generation by FPN-treated hNSCs. Previous studies reported from very low to moderate remyelination capacity from naïve foetal hNSCs after transplantation in several animal models of CNS de/dysmyelination [16], [29] [30]. It was previously shown that the in vivo environment of the acutely demyelinating adult rat spinal cord is insufficient to stimulate the differentiation of immature spinal cord-derived hNSCs [35] and of adult hOPCs [69] to oligodendrocytes, pointing to the crucial role of species-specific signals released at the lesion site in directing and supporting oligodendrocyte migration and differentiation. Consistent with this notion, we have recently reported that hNSCs express the chemokine receptors CXCR4, CCR3, CCR6 and CCR7 and migrate in vitro in response to the cognate human (but not murine) ligands [63]. Also, the presence of significant numbers of engrafted hNSCs still expressing nestin, Sox10 and Olig1 shown in this and in other studies [31], [48] indicates that >2 months might be necessary to allow full maturation of engrafted hNSCs cells, due to their slower timing of differentiation as compared to rodent NSCs [70]. This notion is also supported by data indicating that human glial-committed progenitors take at least 12 weeks to extensively remyelinate the brain of Shiverer mice [5], [6]. In agreement with these findings, we observed a significant engraftment and migration coupled to robust expression of nestin in the majority of engrafted cells two months after transplantation of EF and FPN- hNSCs in the corpus callosum of young Shiverer mouse.

Lack of proper environmental signals and slow timing of hNSC differentiation might indeed provide a key to reconcile the apparent discrepancy between our in vitro and in vivo data. We suggest that hNSCs grown as neurospheres in either EF or FPN contain a large population of cells at the very early steps of the oligodendroglial lineage. FPN priming allows the maintenance of a larger pool of immature oligodendroglial progenitors, likely by a selective more then and inductive mechanism, without significantly modifying their timing of differentiation. Thus, they can progress towards the oligodendroglial lineage only if they encounter the proper sequence of signals in the appropriate time-window. A similar mechanism has been previously proposed to explain the moderate improvement in remyelination ability of hNSCs genetically modified to over express Olig2 as compared to the naïve counterpart [31]. However, the possibility remains that hNSC-derived OPCs are unable to fully differentiate toward a full myelinating phenotype, as suggested for adult OPCs residing in the rodent gray matter [71], [72], either due to intrinsic lineage differences or consequently to a selection/induction by the in vitro conditions.

We observed only few engrafted human cells retaining proliferating ability (expressing Ki67) two months after transplantation. These cells were exclusively localized in the SVZ, where the mouse counterparts undergo the same events. These data are in agreement with previous reports showing the persistence of few proliferating hNSCs after long-term engraftment in the hippocampus, the other major neurogenic region of the adult brain [7].

### Conclusions

Strategies aimed to improve the myelinogenic potential of hNSCs in vivo include direct selection of committed progenitors [3] [5] [45], genetic [31] or epigenetic manipulation [40] [2], [36], [47], [51] of hNSC towards the lineage of interest. We show here that exposure of long-term expanded hNSCs isolated from the developing human brain to defined combinations of pro-oligodendroglial factors results in the generation of large numbers of oligodendroglial cells at the early stages of lineage commitment/differentiation. These cells represent a valuable tool for modelling human oligodendrogenesis in vitro and for drug discovery. Also, the availability of somatic non-immortalized, expandable human neural precursors with low immunogenicity and amenable to in vitro pre-differentiation to oligodendroglial progenitors will facilitate the development and optimization of cell-based therapy for demyelinating disorders. Finally, our results might contribute to the development of efficient protocols to generate oligodendrocyte cultures from patient-specific induced pluripotent stem cells [73], which are emerging as a valuable experimental model to study the pathophysiology of different neurodegenerative diseases.

## Materials and Methods

### Isolation and culture propagation of hNSC

In this study we used two independent NSC lines established and propagated from the diencephalic/telencephalic brain region of two human (h) fetuses at 10.5-week gestational age. The first NSC line used was previously described and characterized [8]; the consent procedure and the ethichs committee that approved the consent procedure were previously reported [8], [12]. The second NSC line was established from brain fetal tissue obtained from Advanced Bioscience Resources, Inc., Alameda, CA, USA. The use of human foetal tissue for the establishment of continuous, non-transformed hNSC lines was approved by the San Raffaele Scientific Institute Ethical committee. This NSC line was kindly provided by Dr. S. Pluchino (Institute of Experimental Neurology, San Raffaele Scientific Institute).

Cells were grown and expanded in mitogen-supplemented serum-free medium (details can be found as File S1) by plating 10^4^ cells/cm^2^ at each subculturing passage in untreated tissue culture flasks. Under these culture conditions both stem and non-stem proliferative progenitors are present in the hNSC population. Cells between passages 18 and 25 were used for the experiments. The two hNSC lines behave similarly in all the experimental conditions tested. Thus, results obtained from the two lines have been pooled for statistical analysis.

### Cultures of hNSC at different stages of maturation

Precursors (highly undifferentiated and proliferating cells) were obtained by dissociating 10-day-old neurospheres, plating single cells (20.000 cells/cm^2^) and growing them for 24 hours in serum-free medium containing EGF and FGF2 (EF medium). Cultures of differentiated cells were obtained by exposing precursors to mitogen-free medium containing either 2% FCS (default differentiation protocol) or different combinations of neurotrophin-3 (NT3), platelet-derived growth factor-AA (PDGF-AA) and thyroid hormone (T3) and growing them for 7–17 days. Details on the composition of the culture media used in different experimental conditions are available as File S1. The extent of neuronal and glial differentiation was assessed by using antibodies against lineage- and stage-specific markers, as described below.

### Migration assay

Migration activity of hNSCs was tested using a Transwell chamber system. Details can be found as File S1.

### RT-PCR

Details on primer sequences and RT-PCR conditions are available in Table S1.

### Immunofluorescence of NSC cultures

Double-labelling immunofluorescence was performed as previously described [25]. Details can be found as File S1. Primary and secondary antibodies used are listed in Table S2.

### Transplantation of hNSCs in animal models of CNS demyelination

#### Cell preparation

Serially passaged 10 day-old neurospheres were collected by centrifugation (1.000 rpm×15 min) and mechanically dissociated. Viable cells were counted by Trypan blue exclusion, washed in PBS and resuspended in PBS + 0.01% DNAse (100.000 cells/µl). Cells were kept in ice until used.

#### Focal lesion

Male NOD SCID (NOD.CB17-*Prkdc* ^scid^/NCrCrl) mice were obtained from Charles Rivers Laboratories International Inc. (Wilmington, MA). Mice were housed in microisolators under sterile conditions and supplied with autoclaved food and water. The use of immunodefcient mice avoided daily immunosuppression.

Two month-old mice were anesthesized with Avertin (Tribromoethanol, Sigma; St. Louis, MO, USA; 1.2%, 200 µl/10 g). Lysolecithin (L-lysophosphatidylcholine; Sigma, St. Louis, MO; 1% in saline; 2 µl) were injected in the right corpus callosum.

Human NSCs grown in EF or in FPN (5 passages) were injected at the lesion site (200.000 cells/2 µl) two days after lysolecithin treatment. Controls received 2 µl of saline. Animals were randomized in three experimental treatment groups: i) Vehicle (n = 2); ii) EF hNSCs (n = 5); iii) FPN hNSCs (n = 5). Eight weeks post-transplantation (pt) mice were deeply anesthetized and intracardially perfused with 0.9% NaCl followed by 4% paraformaldehyde (PFA). Brains were isolated and processed for immunohistochemistry as described below. Protocols regarding animal treatment were approved by the Institutional Committee for the Good Animal Experimentation of the San Raffaele Scientific Institute (IACUC #202 and #314).

### Tissue processing and immunofluorescence analysis

Perfused brains were equilibrated for 24 h in 10–20–30% sucrose in PBS and quick frozen in optimal cutting-temperature compound. Brains were cryostat cut in 16 µm-thick coronal sections. Sections were collected the from the onset of the lateral ventricle (Bregma AP +1.8 mm) to the hippocampal formation (Bregma AP −2 mm) and ordered in 4 series on SuperFrost®Plus glass slides (Menzel-Glaser, Braunschweig, DE); distance between two consecutive sections in each series  = 64 µm. Details on immunofluorescence analysis and on the quantification of cell engraftment are available as File S1. Details on immunofluorescence analysis are available as File S1. Primary and secondary antibodies used are listed in Table S2.

### Statistical analysis

Two way ANOVA followed by Bonferroni's post test was used to compare different treatments, Kruscall-Wallis test followed by Dunn's multiple comparison test or Mann-Whitney test were employed to compare percentages of immunoreactive cells in the different treatments. For growth curves, data were interpolated using a linear regression model and best fitted the following equation: *y*  =  *a* + *b*x, where *y* is the estimated total number of cells, *x* is the time (DIV), *a* is the intercept, and *b* is the slope. The best-fit value, the Std error and the 95% confidence intervals of the slope for each data set were calculated. The slope values were then compared using F test. Statistical significance was accepted with a p value <0.05.

## Supporting Information

Table S1 Primer sequence and RT-PCR conditions.
(0.46 MB DOC)

Table S2 Primary and secondary antibodies used.
(0.06 MB DOC)

Table S3 Absolute numbers (A, B) and averages (A', B') used to calculate percentage values shown in Figure 3D (A and A') and Figure 6B (B and B') in the main text. Values in each row of A and B are the absolute numbers of nuclei (dapi; TOT) and of cells expressing a specific antigen counted in 4–6 randomly selected fields in one coverslip. The corresponding percentages are in brackets. A. Different combinations of antibodies were tested: PDGFRα/SOX10 (2 coverslips/treatment), PDGFRα/OLIG1 (2 coverslips/treatment) and PDGFRα/Ki67 (2 coverslips/treatment). The mean values and the standard error of the mean (SEM) are reported in A'. B. Values are from 3 coverslips in which the anti-PDGFRα antibody was tested. The mean values and the standard error of the mean (SEM) are reported in B'.
(0.07 MB DOC)

Figure S1 Cartoon summarizing the cell type specific quantifications after the different cultivation and priming conditions used in this study. EF hNSCs: cells grown in EF medium FPN hNSCs: EF cells shifted to FPN medium and serially subcultured in this culture condition. Violet arrow indicates hours (h) or days (d) in culture, black and blue arrows indicate the different cultivation protocols, treatments (EF, FPN, FCS, PDGF+T3) are indicated in boxes. Qualitative outcome of the different cultivation protocols are indicated with thick arrows in boxes, arrows up or down in comparison to FCS treatment (white boxes) or to EF (light blue boxes). The reference Figure in which data are shown is indicated in the boxes. Oligodendrocytes: GalCer+; Neurons: TUJ1+; Astrocytes: GFAP+; Proliferating cells: Ki67+, PCNA+; Oligo progenitors: NG2+, Olig1+, PDGFRa+, Sox10+. The percentages of cells (mean±s.e.m).immunoreactive for lineage-specific and proliferation markers in the different treatments and the corresponding Figures in which these data are shown in the tables.
(8.19 MB TIF)

Figure S2 Immunophenotypic signature of hNSC-derived oligodendrocytes. (A–D) Merged immunofluorescence pictures showing NG2+ cells (green), a fraction of them co-expressing GalCer (A) and O4 (B). NG2+ cells do not co-express the astrocytic marker GFAP (C) or the neuronal marker PSA-NCAM (D). NG2, green; GalCer, O4, GFAP, PSA-NCAM, red; yellow-orange indicates merged signal. Quantitative analysis (E) of hNSC cultures exposed to different treatments shows similar numbers of β-tubulin III and PSA-NCAM-expressing cells in the differentiated cultures and an almost complete overlap of the two cell populations (F; merged confocal picture: β-tubulin III, green; PSA-NCAM, red; ToPro3, blue).
(6.32 MB TIF)

Figure S3 FPN treatment allows the enrichment and expansion of an immature oligodendroglial progeny. Representative merged confocal pictures of FPN hNSCs exposed for 10-day to PDGF-AA+T3 showing PDGFRα+ (red) cells co-expressing Olig1(A, green), Sox10+ (B, green) and nestin (C, green). PDGFRα+ cells did not co-express PSA-NCAM (D, green) or GFAP (E, F; green). Nuclei are counterstained with ToPro3 (blue).
(6.64 MB TIF)

Figure S4 Oligodendroglial potential of CTX-derived hNSCs. (A) Cortex (CTX)-derived human NSCs grown in EF medium were plated either in 2% FCS or in the presence of FPN for 7 days. After this priming time, medium was substituted with control medium containing 2% FCS or PDGF-AA+T3 and cultures were grown for additional 10 days. The cell type composition was quantified at the end of the culture period. In FPN-primed cultures the percentages of NG2+ and GalCer+ were increased compared to FCS-treated cultures. Only minor differences were observed in the percenteges of O4+ and TUJ1+ cells. Data are expressed as the mean±SEM, n = 2 independent experiments, 1–5 replicates/experiment. Comparisons between the different treatments were performed for individual antigens using Kruscal-Wallis test followed by Dunn's multiple comparison test. **p<0.01 and *p<0.05 vs FCS. (B) CTX hNSCs in FPN medium significantly reduced their growth rate, still maintaining their self-renewing capacity for at least 5 subculturing passages. Data are the mean±SEM of three independent experiments. Data were interpolated using a linear regression model and best fitted the following equation: y  =  a + bx, where y is the LOG10 of the total number of cells, x is the time (DIV), a is the intercept, and b is the slope. Values of b±SEM are 0.02237±0.00216 and 0.01555±0.00168 for EF and FPN, respectively, **p = 0.0307. (C–F) Representative immufluorescence pictures of neurons (TUJ1, red) and astrocytes (GFAP, green; C), and oligodendrocytes (O4 and GalCer, red; NG2, green; D–F) in FPN-primed culture (FPN>PDGF+T3). Note the mature morphology of GalCer-expressing cells.
(5.27 MB TIF)

Figure S5 Human NSC transplantation in Shiverer mice. (A) Two months after unilateral injection in the right corpus callosum of post-natal day 20 Shiverer mice, LV.eGFP-T hNSCs are migrated along the corpus callosum in the controlateral hemisphere and show morphology and immunogenic features of undifferentiated neural cells. GFP, green; nestin, red; blue, dapi; yellow-orange, merged signal. Individual confocal pictures (10× magnification, 7 pictures for each channel and for the merged series) were composed using the Photomerge tool of Adobe Photoshop CS3. Higher magnification of the left (B) and right (C) hemisphere showing co-localization of nestin (red) and GFP (green) immunoreactivity. Right, injected site; left, controlateral site. LV, lateral ventricles.
(4.73 MB TIF)

File S1 (0.04 MB DOC)
