# Supplementary material for: Robust Generation of Oligodendrocyte Progenitors from Human Neural Stem Cells and Engraftment in Experimental Demyelination Models in Mice
Source: PLoS One. 2010 Apr 12;5(4):e10145. doi: 10.1371/journal.pone.0010145 (PMC2853578; doi:10.1371/journal.pone.0010145)
Supplement: Table S1 — Primer sequence and RT-PCR conditions. (0.46 MB DOC) [file pone.0010145.s001.doc]

**Table S1. Primer sequence and RT-PCR conditions**
