# Supplementary material for: Robust Generation of Oligodendrocyte Progenitors from Human Neural Stem Cells and Engraftment in Experimental Demyelination Models in Mice
Source: PLoS One. 2010 Apr 12;5(4):e10145. doi: 10.1371/journal.pone.0010145 (PMC2853578; doi:10.1371/journal.pone.0010145)
Supplement: Table S2 — Primary and secondary antibodies used. (0.06 MB DOC) [file pone.0010145.s002.doc]

**Table S2**. Primary and secondary antibodies used

| **Primary antibodies** | **Source and antibody type** | **Dilution used** | |
| --- | --- | --- | --- |
| *Antigen* |  | *Cells* | *Tissues* |
| βIV-tubulin | Sigma (St. Louis, MO, USA)  mouse monoclonal IgG1, #T7941 |  | 1:500 |
| Galactocerebroside (GalC) | Chemicon (Millipore)  mouse monoclonal IgG3, #MAB342 | 1:200 |  |
| Green Fluorescence Protein (GFP) | Molecular probes (Carlsbad, CA, USA)  rabbit polyclonal, #A-11122 | 1:500 |  |
| Glial fibrillary acidic protein (GFAP) | Dako, (Glostrup, Denmark)  rabbit polyclonal, #ZO334 | 1:500 | 1:400 |
| Glutamine Sinthase (GS) | Chemicon (Millipore)  Mouse monoclonal IgG2a, #MAB302 |  | 1:1000 |
| Glutathione-S-transferase π (GST-π) | MBL International (Woburn, MA, USA)  rabbit poyclonal #312 |  | 1:5.000 |
| Human Mitochondria | Chemicon (Millipore)  mouse monoclonal IgG1, #MAB1273 |  | 1:100 |
| Human Nuclei | Chemicon (Millipore)  mouse monoclonal IgG1, #MAB1281 |  | 1:100 |
| Ki67 | Novocastra (Newcastle, UK)  mouse monoclonal NCL-ki67MM1  rabbit polyclonal NCL-Ki76p | 1:1.000 | 1:1.000 |
| MBP | Chemicon (Millipore)  mouse monoclonal IgG, #MAB386 | 1:300 | 1:300 |
| hNestin | Chemicon (Millipore)(Billera, MA, USA)  rabbit polyclonal, MAB342 | 1:200 | 1:500 |
| Neuronal Class III β-Tubulin | Babco (Richmond, CA, USA), mouse monoclonal, IgG2a, clone TUJ1, #MMS-435P and rabbit polyclonal, #PRB-435P | 1:500 | 1:500 |
| NG2 chondroitin sulfate proteoglycan | Chemicon (Millipore)  rabbit polyclonal, #AB5320 | 1:300 | 1:300 |
| Olig1 | Chemicon (Millipore)  rabbit polyclonal, #AB5591 | 1:200 | 1:100 |
| Oligodendrocyte marker O4 | Chemicon (Millipore)  mouse monoclonal IgM, clone 81. Commonly referred to in the literature as monoclonal antibody O4, #MAB345 | 1:100 |  |
| PCNA | Sigma (St. Louis, MO, USA)  mouse monoclonal IgG2a, #P8825 | 1:100 |  |
| PDGFR-α | BD Pharmingen (Franklin Lakes, NJ, USA)  BD140A | 1:150 |  |
| PSA-NCAM | Valbiotech (Paris, France)  Mouse monoclonal IgM, #ABC0019 | 1:1.000 | 1:2.000 |
| Sox10 | Immunological science (Roma, IT)  rabbit polyclonal, #AB10875 | 1:250 | 1:200 |
|  |  |  |  |
| **Secondary antibodies** |  |  |  |
| Alexa 488-conjugated | Molecular probes (Carlsbad, CA, USA)  goat anti-mouse, #A11001 o and goat anti-rabbit IgG, #A11008 | 1:2.000 | 1:1.000 |
| Alexa 546-conjugated | Molecular probes (Carlsbad, CA, USA)  goat anti-mouse, #A11003 o and goat anti-rabbit IgG, #A11010 | 1:2.000 | 1:2.000 |
| Cy3-conjugated | Jackson immunoresearch (Newmarket, Suffolk, UK)  Goat anti-mouse, #115165062 o and goat anti-rabbit IgG, #111165144 | 1:2000 | 1:2000 |
