# Supplementary material for: Robust Generation of Oligodendrocyte Progenitors from Human Neural Stem Cells and Engraftment in Experimental Demyelination Models in Mice
Source: PLoS One. 2010 Apr 12;5(4):e10145. doi: 10.1371/journal.pone.0010145 (PMC2853578; doi:10.1371/journal.pone.0010145)
Supplement: Table S3 — Absolute numbers (A, B) and averages (A', B') used to calculate percentage values shown in Figure 3D (A and A') and Figure 6B (B and B') in the main text. Values in each row of A and B are the absolute numbers of nuclei (dapi; TOT) and of cells expressing a specific antigen counted in 4–6 randomly selected fields in one coverslip. The corresponding percentages are in brackets. A. Different combinations of antibodies were tested: PDGFRα/SOX10 (2 coverslips/treatment), PDGFRα/OLIG1 (2 coverslips/treatment) and PDGFRα/Ki67 (2 coverslips/treatment). The mean values and the standard error of the mean (SEM) are reported in A'. B. Values are from 3 coverslips in which the anti-PDGFRα antibody was tested. The mean values and the standard error of the mean (SEM) are reported in B'. (0.07 MB DOC) [file pone.0010145.s003.doc]

**Table S4. Absolute numbers (A, B) and averages (A’, B’) used to calculate percentage values shown in Figure 3D (A and A’) and Figure 6B (B and B’) in the main text.**

| **A** | **total number of cells (%)** | | | | |  |  |  |  |  |  |
| --- | --- | --- | --- | --- | --- | --- | --- | --- | --- | --- | --- |
| **Treatments** | **TOT** | **PDGFRα** | **SOX10** |  | **TOT** | **PDGFRα** | **OLIG1** |  | **TOT** | **PDGFRα** | **Ki67** |
| FCS | 418 | 20 (4.78) | 2 (0.47) |  | 303 | 28 (9.24) | 186 (61.38) |  | 361 | 48 (13.29) | 10 (2.77) |
| FPN>FCS | 378 | 30 (7.93) | 6 (1.58) |  | 294 | 56 (19.04) | 206 (70.07) |  | 367 | 107 (29.15) | 53 (14.44) |
| FPN>PDGF+T3 | 149 | 19 (12.75) | 3 (2.01) |  | 138 | 46 (33.33) | 107 (77.53) |  | 182 | 75 (41.21) | 45 (24.72) |
|  |  |  |  |  |  |  |  |  |  |  |  |
| **Treatments** | **TOT** | **PDGFRα** | **SOX10** |  | **TOT** | **PDGFRα** | **OLIG1** |  | **TOT** | **PDGFRα** | **KI67** |
| FCS | 349 | 7 (2.00) | 3 (0.86) |  | 144 | 8 (5.55) | 61 (42.36) |  | 461 | 29 (6.29) | 4 (0.86) |
| FPN>FCS | 440 | 20 (4.54) | 9 (2.05) |  | 168 | 26 (15.47) | 122 (72.61) |  | 301 | 38 (12.62) | 29 (9.63) |
| FPN>PDGF+T3 | 186 | 17 (9.13) | 19 (10.21) |  | 88 | 31 (35.22) | 77 (87.5) |  | 164 | 67 (40.85) | 36 (21.95) |

| **A'** | **total nr of cells** | |  | **PDGFRα** |  |  | **Sox10** |  |  | **Olig1** |  |  | **Ki67** |  |  |
| --- | --- | --- | --- | --- | --- | --- | --- | --- | --- | --- | --- | --- | --- | --- | --- |
| **Treatments** | *mean* | *SEM* | *n* | *mean* | *SEM* | *n* | *mean* | *SEM* | *n* | *mean* | *SEM* | *n* | *mean* | *SEM* | *n* |
| FCS>FCS | 339.3 | 45.1 | 6 | 23.3 | 6.25 | 6 | 2.5 | 0.5 | 2 | 123.5 | 62.5 | 2 | 7 | 3 | 2 |
| FPN>FCS | 324.6 | 38.2 | 6 | 46.1 | 13.18 | 6 | 7.5 | 1.5 | 2 | 164 | 42 | 2 | 41 | 12 | 2 |
| FCS>PDGF+T3 | 151.1 | 14.7 | 6 | 42.5 | 10.03 | 6 | 11 | 8 | 2 | 92 | 15 | 2 | 40.5 | 4.5 | 2 |

| **B** | **total number of cells (%)** | | | |  |  |  |  |
| --- | --- | --- | --- | --- | --- | --- | --- | --- |
| **Treatments** | **TOT** | **PDGFRα** |  | **TOT** | **PDGFRα** |  | **TOT** | **PDGFRα** |
| EF | 294 | 31 (10.54) |  | 285 | 53 (18.6) |  | 252 | 38 (15.1) |
| FPN | 186 | 25 (13.4) |  | 144 | 55 (38.2) |  | 204 | 62 (30.4) |

| **B'** | **total nr of cells** | |  |  | **PDGFRα** |  |  |
| --- | --- | --- | --- | --- | --- | --- | --- |
| **Treatments** | mean | SEM | n |  | mean | SEM | n |
| EF | 277 | 12.76 | 3 |  | 40.7 | 6.5 | 3 |
| FPN | 178 | 17.7 | 3 |  | 47.7 | 11 | 3 |
