# Supplementary figures and images for: Robust Generation of Oligodendrocyte Progenitors from Human Neural Stem Cells and Engraftment in Experimental Demyelination Models in Mice
Source: PLoS One. 2010 Apr 12;5(4):e10145. doi: 10.1371/journal.pone.0010145 (PMC2853578; doi:10.1371/journal.pone.0010145)

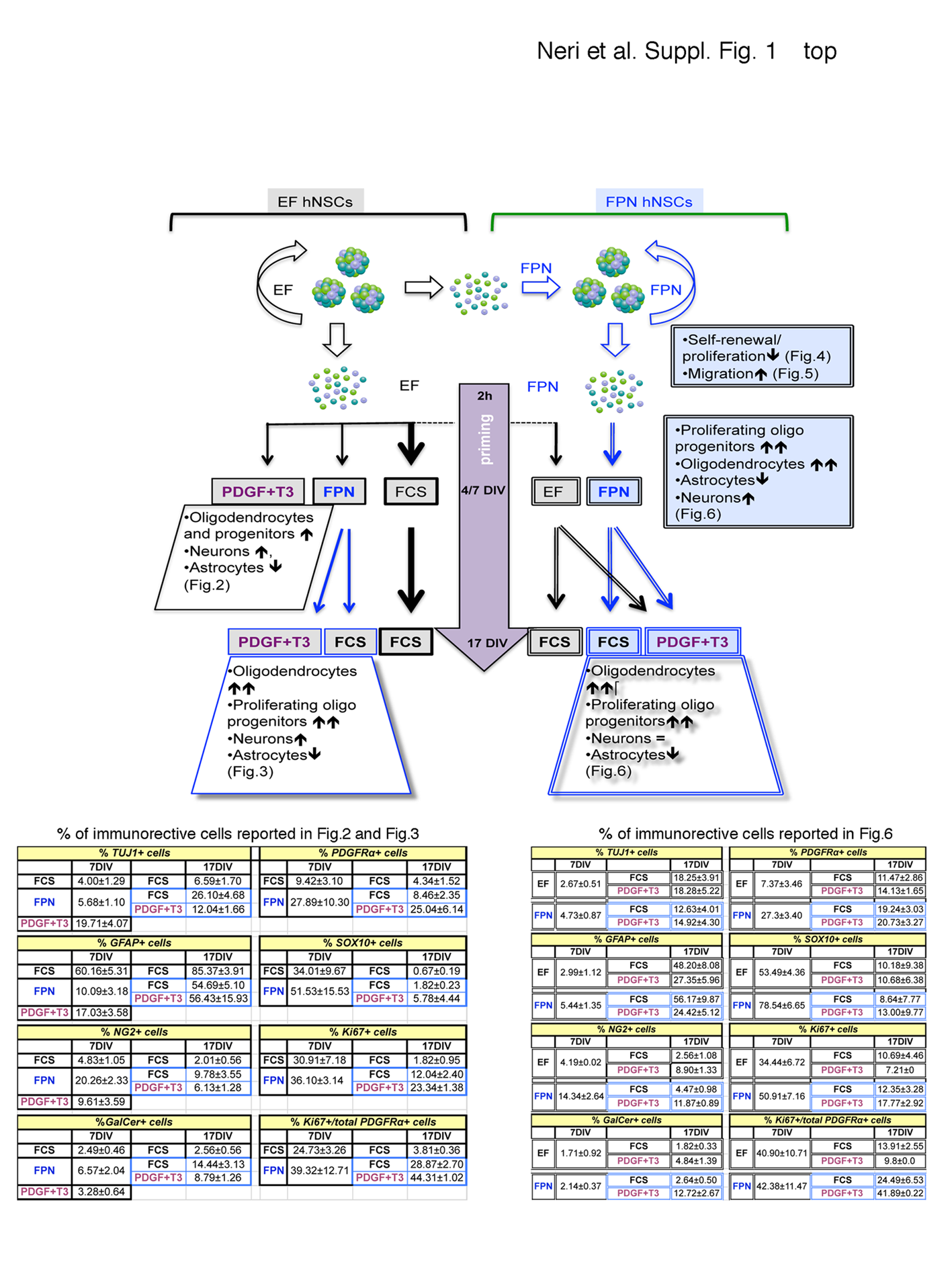

Supplement: Figure S1 — Cartoon summarizing the cell type specific quantifications after the different cultivation and priming conditions used in this study. EF hNSCs: cells grown in EF medium FPN hNSCs: EF cells shifted to FPN medium and serially subcultured in this culture condition. Violet arrow indicates hours (h) or days (d) in culture, black and blue arrows indicate the different cultivation protocols, treatments (EF, FPN, FCS, PDGF+T3) are indicated in boxes. Qualitative outcome of the different cultivation protocols are indicated with thick arrows in boxes, arrows up or down in comparison to FCS treatment (white boxes) or to EF (light blue boxes). The reference Figure in which data are shown is indicated in the boxes. Oligodendrocytes: GalCer+; Neurons: TUJ1+; Astrocytes: GFAP+; Proliferating cells: Ki67+, PCNA+; Oligo progenitors: NG2+, Olig1+, PDGFRa+, Sox10+. The percentages of cells (mean±s.e.m).immunoreactive for lineage-specific and proliferation markers in the different treatments and the corresponding Figures in which these data are shown in the tables. (8.19 MB TIF) [file pone.0010145.s004.tif]

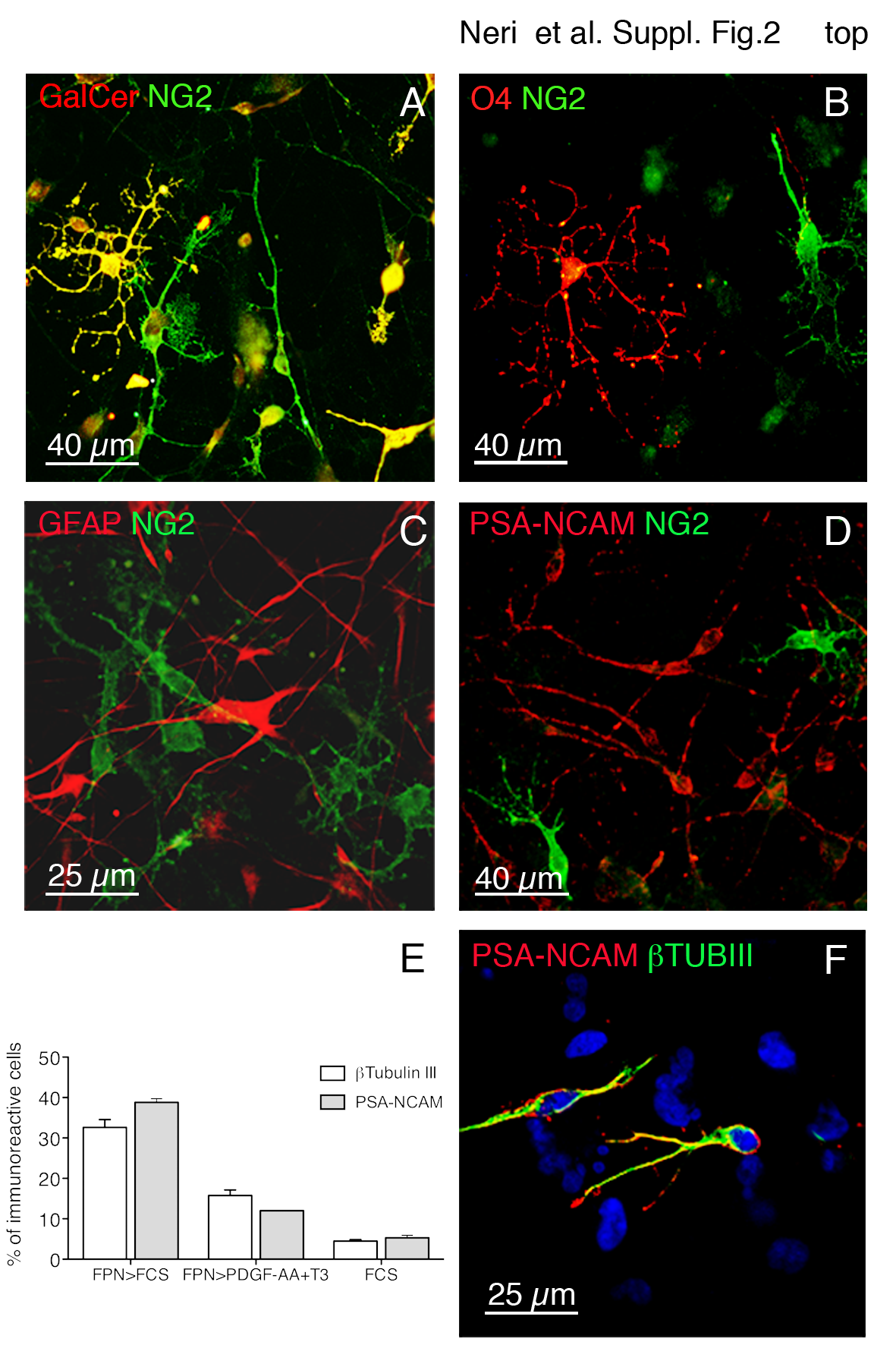

Supplement: Figure S2 — Immunophenotypic signature of hNSC-derived oligodendrocytes. (A–D) Merged immunofluorescence pictures showing NG2+ cells (green), a fraction of them co-expressing GalCer (A) and O4 (B). NG2+ cells do not co-express the astrocytic marker GFAP (C) or the neuronal marker PSA-NCAM (D). NG2, green; GalCer, O4, GFAP, PSA-NCAM, red; yellow-orange indicates merged signal. Quantitative analysis (E) of hNSC cultures exposed to different treatments shows similar numbers of β-tubulin III and PSA-NCAM-expressing cells in the differentiated cultures and an almost complete overlap of the two cell populations (F; merged confocal picture: β-tubulin III, green; PSA-NCAM, red; ToPro3, blue). (6.32 MB TIF) [file pone.0010145.s005.tif]

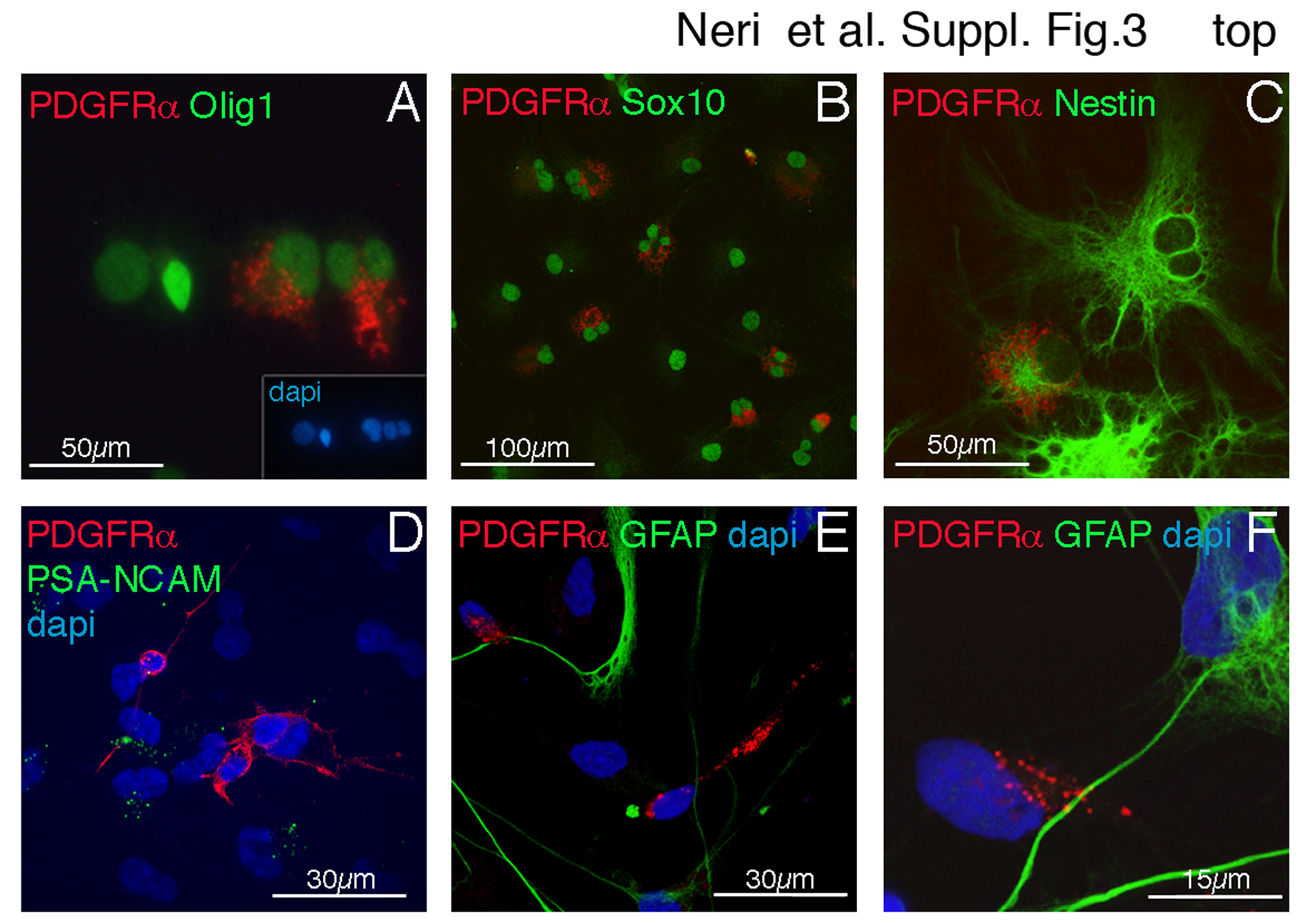

Supplement: Figure S3 — FPN treatment allows the enrichment and expansion of an immature oligodendroglial progeny. Representative merged confocal pictures of FPN hNSCs exposed for 10-day to PDGF-AA+T3 showing PDGFRα+ (red) cells co-expressing Olig1(A, green), Sox10+ (B, green) and nestin (C, green). PDGFRα+ cells did not co-express PSA-NCAM (D, green) or GFAP (E, F; green). Nuclei are counterstained with ToPro3 (blue). (6.64 MB TIF) [file pone.0010145.s006.tif]

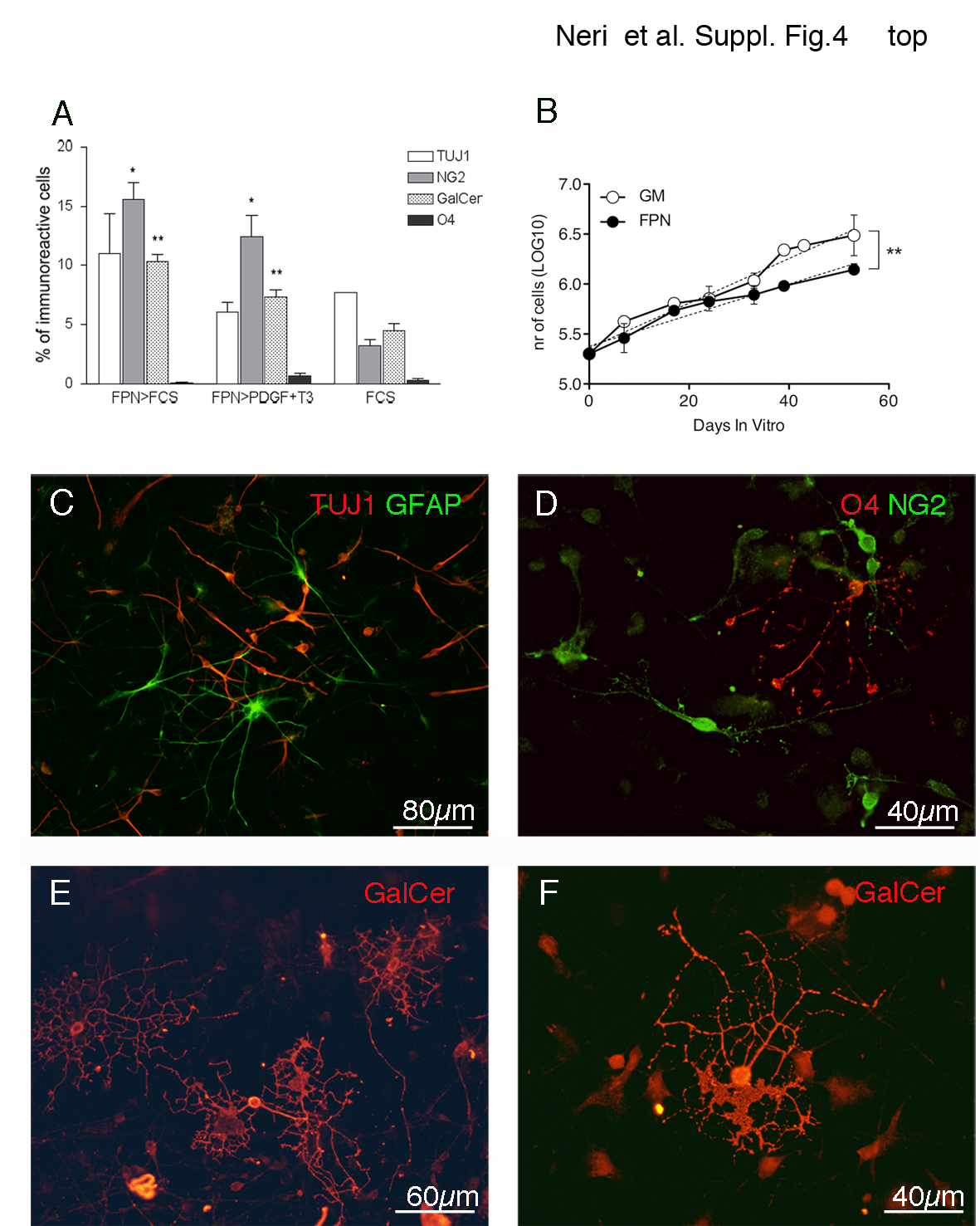

Supplement: Figure S4 — Oligodendroglial potential of CTX-derived hNSCs. (A) Cortex (CTX)-derived human NSCs grown in EF medium were plated either in 2% FCS or in the presence of FPN for 7 days. After this priming time, medium was substituted with control medium containing 2% FCS or PDGF-AA+T3 and cultures were grown for additional 10 days. The cell type composition was quantified at the end of the culture period. In FPN-primed cultures the percentages of NG2+ and GalCer+ were increased compared to FCS-treated cultures. Only minor differences were observed in the percenteges of O4+ and TUJ1+ cells. Data are expressed as the mean±SEM, n = 2 independent experiments, 1–5 replicates/experiment. Comparisons between the different treatments were performed for individual antigens using Kruscal-Wallis test followed by Dunn's multiple comparison test. **p<0.01 and *p<0.05 vs FCS. (B) CTX hNSCs in FPN medium significantly reduced their growth rate, still maintaining their self-renewing capacity for at least 5 subculturing passages. Data are the mean±SEM of three independent experiments. Data were interpolated using a linear regression model and best fitted the following equation: y = a + bx, where y is the LOG10 of the total number of cells, x is the time (DIV), a is the intercept, and b is the slope. Values of b±SEM are 0.02237±0.00216 and 0.01555±0.00168 for EF and FPN, respectively, **p = 0.0307. (C–F) Representative immufluorescence pictures of neurons (TUJ1, red) and astrocytes (GFAP, green; C), and oligodendrocytes (O4 and GalCer, red; NG2, green; D–F) in FPN-primed culture (FPN>PDGF+T3). Note the mature morphology of GalCer-expressing cells. (5.27 MB TIF) [file pone.0010145.s007.tif]

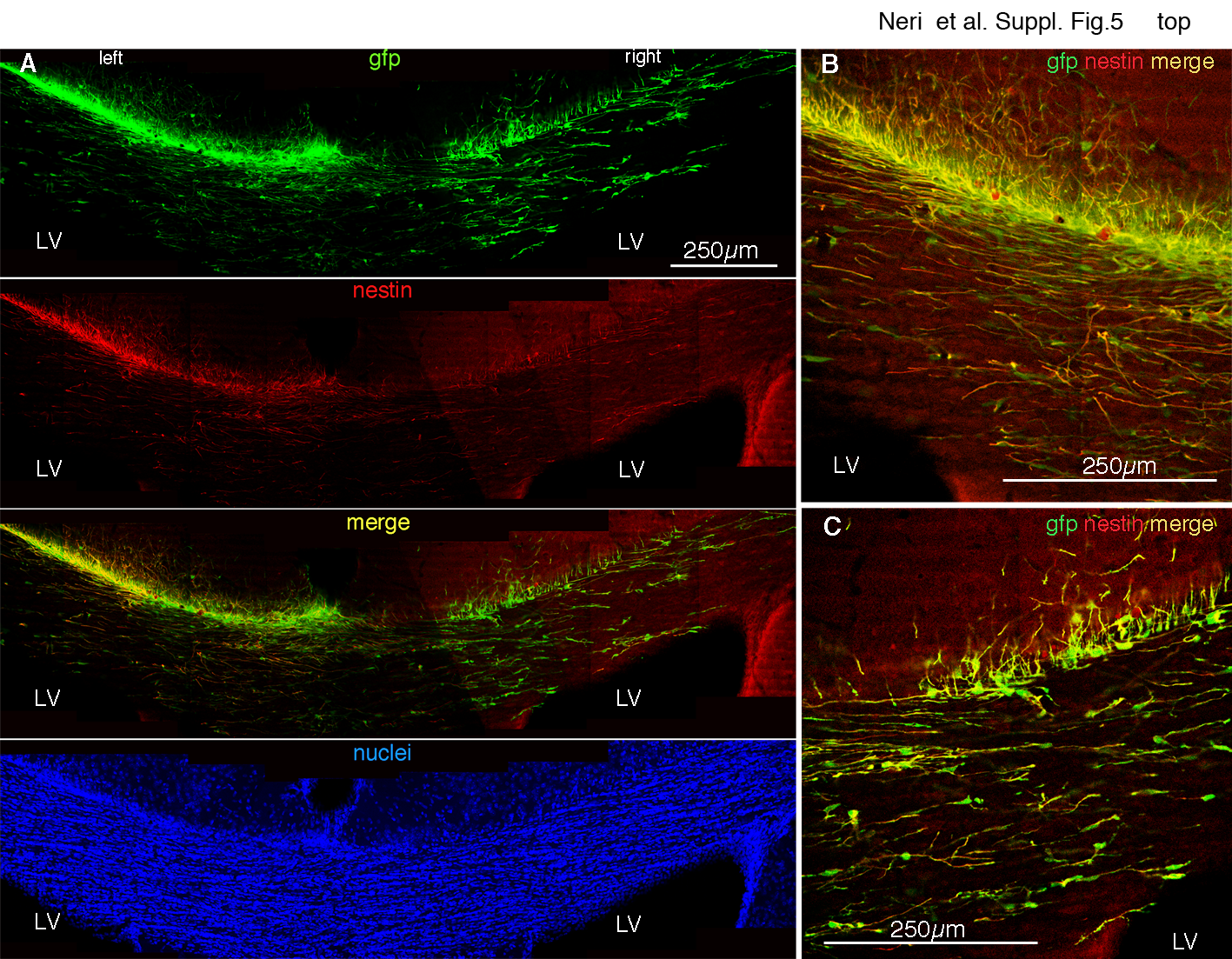

Supplement: Figure S5 — Human NSC transplantation in Shiverer mice. (A) Two months after unilateral injection in the right corpus callosum of post-natal day 20 Shiverer mice, LV.eGFP-T hNSCs are migrated along the corpus callosum in the controlateral hemisphere and show morphology and immunogenic features of undifferentiated neural cells. GFP, green; nestin, red; blue, dapi; yellow-orange, merged signal. Individual confocal pictures (10× magnification, 7 pictures for each channel and for the merged series) were composed using the Photomerge tool of Adobe Photoshop CS3. Higher magnification of the left (B) and right (C) hemisphere showing co-localization of nestin (red) and GFP (green) immunoreactivity. Right, injected site; left, controlateral site. LV, lateral ventricles. (4.73 MB TIF) [file pone.0010145.s008.tif]
