# Supplementary material for: Robust Generation of Oligodendrocyte Progenitors from Human Neural Stem Cells and Engraftment in Experimental Demyelination Models in Mice
Source: PLoS One. 2010 Apr 12;5(4):e10145. doi: 10.1371/journal.pone.0010145 (PMC2853578; doi:10.1371/journal.pone.0010145)
Supplement: File S1 — (0.04 MB DOC) [file pone.0010145.s009.doc]

**Supporting information file S1**

**Methods**

***Composition of culture media used in different experimental conditions***

Control medium: DMEM/F12 medium (1/1 v/v) containing L-glutamine (2mM), glucose (0.6%), putrescine (9.6 µg/ml), insulin (0.025 mg/ml), progesteron (6.3 ng/ml), apo-transferrin (0.1 mg/ml), sodium selenite (5.2 ng/ml). All reagents from Sigma, MO, USA.

This is the basal medium used to prepare the growth and differentiation media.

EF medium: control medium containing EGF (20 ng/ml) and FGF2 (10 ng/ml).

This medium is used to isolate, grow and expand NSCs.

FPN medium: PDGF-AA (20 ng/ml)+FGF2 (10 ng/ml) +NT3 (20 ng/ml). This medium is used for priming experiments and for expansion of hNSC-derived oligodendroglial progenitors.

Differentiating media: control medium containing

- 2% fetal calf serum (FCS)
- PDGF-AA (20 ng/ml)+T3 (40 ng/ml)

Human recombinant EGF, FGF2, PDGF-AA, NT3 are from Peprotech, NJ, USA. T3 is purchased from Sigma. These media are used to generate differentiated cultures on which a quantitative analysis of the different cell types (neurons, astrocytes and oligodendrocytes) was carried out**.**

***RT-PCR*.**

Total RNA was extracted using the RNeasy Mini kit (Qiagen, Valencia, CA). cDNA was obtained using Superscript RNase H– Reverse Transcriptase (Invitrogen, Paisley, UK). PCR was carried out using Taq DNA Polymerase (Qiagen) according to manufacturer’s protocol. All cDNAs used as templates were previously normalized throughout β-Actin RT-PCR.

The PCR products were separated on a 2% agarose gel at 120V and visualized by ethidium bromide.

***Immunohistochemistry and immunofluorescence analysis.***

### *Cell cultures.* Cultures were fixed in 4 % paraformaldehyde, rinsed with PBS and incubated for 120 min at 37°C in PBS containing 10% normal goat serum (NGS), 0.1% Triton X-100 (omitted when antibodies against membrane-bound antigens were used) and appropriate primary antibodies. After washing cells were reacted for 1 hr at room temperature (RT) with the appropriate secondary antibodies. Samples were rinsed three times with PBS, once with distilled water, and mounted with Fluorsave (Calbiochem, La Jolla, CA). A list of primary and secondary antibodies is available in Supplementary Table 2. Negative controls included hNSC cultures in which primary antibodies were omitted. In this case, no positive signal was observed. Nuclei were counterstained with 4,6-diamine-2-phenylindole dihydrochloride (DAPI; Roche, Indianapolis, IN, USA; 50 μg/ml in PBS) or with ToPro3 (Molecular Probes, Carlsbad, CA, USA) for 10 min at RT. Immunoreactive cells were counted in 6 fields in each sample (>500 cells/sample; 2-4 independent experiments) and expressed as a percentage of the total number of nuclei.

*Tissue sections.* Tissue sections were blocked in PBS/10% normal goat serum/0.3% Triton X-100 for 1 hour, incubated overnight at 4°C with primary antibodies for the detection of human-specific antigens and lineage-specific markers, washed 3 times with PBS and then incubated at room temperature for 2h with the appropriate secondary antibodies in PBS/1% normal goat serum. Sections were then washed in PBS and cover-slipped in permanent mounting medium.

***Migration assay.***

Migration activity of hNSCs was tested by using a 24-well plate Transwell chamber system. HNSCs (20.000 cells) were plated on Matrigel-coated membranes (8 µm diameter pores) in 100µl of control media (upper chamber). The lower chambers were filled with 600µl of different media: PDGF-AA+T3 (20 and 40 ng/ml, respectively), FPN or EF. After 72h the upper chambers were removed and washed in PBS. Cells attached to the upper face of the membranes were gently removed using a cotton scrub. Membranes were then fixed in methanol, processed for hematoxylin/eosin staining and mounted on glass slides. Cells were counted at the microscope (bright field, 40x magnification) in 10 random fields/filter. Migration activity was calculated as the mean number of cells/field and expressed as the fold-increase to basal conditions (control medium). Data are expressed as the mean ± SEM and derive from 2-4 independent experiments, 2 replicates for each experiment.

***Quantification of cell engraftment***

All the sections were scored for the presence of human-derived cells using the 3 antibodies available (anti-hNu, anti-hNest and anti-hMit). hNu^+^ cells were counted in all the sections of one series in each of the 5 mice/group, the sum was multiplied by 4 in order to have an estimate of the total number of engrafted cells/brain. The distance between the most rostral and the most caudal section in which human cells were detected gave an estimate of cell dispersion (expressed in µm).

Samples (cell cultures and tissues) were visualized with: i) Nikon Eclipse E600 epifluorescence microscope. Images were acquired using a Nikon DMX 1200 digital camera and ACT-1 acquisition software (Nikon); ii) Zeiss Axioskop2 microscope using double laser confocal microscopy with Zeiss Plan-Neofluar objective lens (Zeiss, Arese, Italy). Images were acquired using a Radiance 2100 camera (Bio-Rad, Segrate, Italy) and LaserSharp 2000 acquisition software (Bio-Rad). Confocal images were imported into Adobe Photoshop CS3 (USA) and adjusted for brightness and contrast.

***Human NSC transplant in Shiverer mice.***

Shiverer mice (C3Fe.SWV-*Mbp^shi+^*) were obtained from Jackson Laboratory (Bar Harbor, ME, http://www.jax.org). Mice were kept in heterozygosis in the animal facility at the San Raffaele Institute. For the transplant experiment, 20 day-old homozygous mice were selected according to the presence of the shivering phenotype.

Serially passaged hNSCs grown in EF medium were transduced by means of lentiviral vectors (LV) encoding for the eGFP cDNA ^1, 2^, as previously described ^3^. After one subculturing passage half of the LV.eGFP-transduced(T) hNPCs were shifted in FPN medium and subcultured twice before being transplanted.

EF- or FPN-hNPCs (200.000 cells/2µl) were injected in the right corpus callosum (mm from bregma: AP +1, ML +1.1, DV –2.2) of post-natal day 20 mice (Jackson Laboratories; n=3 for each group). Mice were treated daily with Cyclosporin (Sandimmun, Sandoz, 10 mg/kg i.p.) from the day before transplantation until the time of sacrifice (2, 3 and 4 months p.t). Mice were intracardially perfused with saline and 4% PFA. Brains were collected and processed for the detection of engrafted cells by means of immunofluorescence analysis using an anti-GFP antibody as described above

**References**

1. Naldini, L. Lentiviruses as gene transfer agents for delivery to non-dividing cells. *Curr Opin Biotechnol* **9**, 457-463 (1998).

2. Consiglio, A. et al. Robust in vivo gene transfer into adult mammalian neural stem cells by lentiviral vectors. *Proc Natl Acad Sci U S A* **101**, 14835-14840 (2004).

3. Pluchino, S. et al. Human neural stem cells ameliorate autoimmune encephalomyelitis in non-human primates. *Ann Neurol* **66**, 343-354 (2009).
